# Reduced NK cell frequency in older patients with evolving fractures is linked to a distinct inflammatory cytokine profile

**DOI:** 10.1186/s12979-026-00585-5

**Published:** 2026-07-24

**Authors:** Julia P. Moch, Jolane Kappes, Rodrigo Gutierrez Jauregui, Hagen Schmaus, Malou-Sophie Dietrich, Daniel A. Thies, Lennart M. Roesner, Thomas Werfel, Reinhold Förster, Dorothee von Witzendorff, Jennifer Debarry, Marcel Winkelmann, Swantje Oberthür, Jan-Dierk Clausen, Manfred Gogol, Markus Cornberg, Anke R. M. Kraft, Christian Niehaus

**Affiliations:** 1 Centre for Individualised Infection Medicine (CiiM), a joint venture between the Helmholtz Centre for Infection Research (HZI) and Hannover Medical School (MHH), Feodor-Lynen-Straße 7, Hannover, Germany; 2 Department of Trauma Surgery, Hannover Medical School, Hannover, Germany; 3 Twincore, Centre for Experimental and Clinical Infection Research, a joint venture between the Helmholtz Centre for Infection Research (HZI) and the Hannover Medical School, Hannover, Germany; 4 Department of Gastroenterology, Hepatology, Infectious Diseases and Endocrinology, Hannover Medical School, Carl-Neuberg-Straße 1, Hannover, Germany; 5 Cluster of Excellence RESIST (EXC 2155), Hannover Medical School, Hannover, Germany; 6 Institute of Immunology, Hannover Medical School, Hannover, Germany; 7 Department of Dermatology and Allergy, Hannover Medical School, Hannover, Germany; 8 German Center for Infection Research (DZIF), Partner-Site Hannover-Braunschweig, Hannover, Germany

**Keywords:** Age-associated immune dysfunction, Fractures, Frailty, NK cells, Soluble immune mediators

## Abstract

**Background:**

Proximal hip fractures represent a profound acute physiological stress in older adults and are often followed by infections, delayed recovery, and functional decline. These complications occur more frequently in frail individuals with reduced physiological resilience and impaired immune responses. As natural killer (NK) cells are central to early immune defense, we aimed to define how acute fracture, hospitalization and frailty shape NK cell homeostasis and function in older patients.

**Methods:**

We conducted a prospective study including older patients (> 65 years) with acute fractures (SMART cohort; n = 103) and compared them to matched healthy older people from the RESIST senior individuals (SI) cohort (n = 550). A subset of SMART patients (n = 55) was longitudinally followed-up post-surgery. NK cell frequency, phenotype and function were investigated using multiparametric flow cytometry, and plasma soluble immune mediators (SIMs) were analyzed.

**Results:**

SMART patients were clinically frailer than matched SI individuals, as indicated by reduced grip strength and lower Barthel scores, and exhibited an inflammatory state with elevated CRP levels and leukocyte counts. NK cell frequencies were significantly reduced in SMART patients and inversely correlated with grip strength and systemic inflammation. Furthermore, NK cells from SMART patients showed a distinct immune phenotype and altered chemokine receptor expression compared with SI individuals. Of note, differences between frail and non-frail patients within the SMART cohort were modest and substantially smaller than those observed between SMART patients and SI individuals. Functionally, frail patients displayed reduced baseline expression of cytotoxic molecules, whereas cytokine-induced NK cell responses were preserved. Furthermore, longitudinal analyses revealed stable NK cell frequencies but surgical intervention remodeled NK cell subset distribution and marker expression.

**Conclusions:**

In conclusion, our findings indicate that NK cell alterations in older patients with fractures are likely driven by the combined impact of acute injury, hospitalization and surgery rather than by frailty alone.

**Supplementary Information:**

The online version contains supplementary material available at 10.1186/s12979-026-00585-5.

## Background

Aging is a dynamic process that ultimately leads to increased vulnerability to acute and chronic diseases, disability and mortality [1]. Globally, populations are aging rapidly, with a continuous rise in the proportion of individuals aged 60 years and older. In parallel, the number of centenarians is projected to increase substantially by 2050, underscoring the growing relevance of age-related health challenges [2]. A hallmark of aging is the progressive dysregulation of immune function. This includes a decline in immune competence, commonly referred to as immunosenescence, and the emergence of chronic low-grade systemic inflammation, termed inflammaging [3]. Together, these immune alterations contribute to increased susceptibility to infections, impaired responses to surgical stress, and delayed recovery, resulting in higher rates of postoperative complications and morbidity in older patients [4]. One of the most clinically relevant consequences of aging is frailty, a condition characterized by diminished physiological reserve and reduced resistance to internal and external stressors [5–7]. Indeed, recent studies have shown that frail patients have an increased risk of postoperative complications, including infections, wound healing, delayed recovery after surgical interventions, and higher mortality [7–9]. However, the biological mechanisms underlying this increased vulnerability remain incompletely understood. Thus, there is an urgent need to better understand why these patients are at risk for poor outcomes.

Although age-associated immune changes have been extensively studied, considerably less is known about how immune dysregulation differs between robust and frail older individuals [3, 10]. Not all older individuals develop frailty, suggesting substantial heterogeneity in immune aging trajectories. Importantly, frailty is often conceptualized as a static baseline condition, whereas the extent to which acute physiological stressors such as trauma or hospitalization reshape immune function beyond pre-existing frailty remains poorly defined. Understanding the immunological features that distinguish frail from non-frail individuals and how these features are modulated by acute stress may help identify patients at risk for poor postoperative outcomes and guide the development of targeted preventive or therapeutic strategies [11].

Among lymphocyte subsets, natural killer (NK) cells have been previously linked to frailty in older adults [12]. NK cells are innate immune cells and represent 5–20% of circulating lymphocytes, that serve as the first line of defense and are involved in antiviral and anticancer immune responses [13–15]. Upon activation, NK cells can respond rapidly with the production of proinflammatory and cytotoxic molecules [16]. Emerging evidence suggests that NK cell homeostasis is particularly relevant in older adults, as lower NK cell counts have been associated with increased mortality [17]. Moreover, impaired NK cell cytotoxicity has been reported in older individuals with chronic diseases, infections, or malignancies and correlates with poorer clinical outcomes [18]. These observations position NK cells as potential key mediators linking immune aging, systemic inflammation, and vulnerability to acute physiological stress.

In this study, we aimed to comprehensively investigate the immune compartment in older patients hospitalized with acute bone fractures and unveil differences between clinically vulnerable and healthy senior individuals. Specifically, we sought to determine whether NK cell alterations in this setting primarily reflect baseline frailty or are predominantly driven by the acute injury and hospitalization itself. Therefore, we established a prospective cohort of 103 older patients admitted to the Trauma Department of Hannover Medical School due to acute bone fractures (SMART cohort (“E**s**tablishment of a cohort for reducing postoperative infections through customized therapies in elderly patients”)). NK cell phenotype and function, as well as soluble immune mediators (SIMs) in peripheral blood, were analyzed and compared with healthy individuals from the Resolving Infection Susceptibility (RESIST) Senior Individuals (SI) cohort [19]. Moreover, frail and non-frail patients within the SMART cohort were analyzed to compare NK cell phenotype and function for intra-cohort comparisons. In addition, longitudinal analyses were performed to investigate postoperative changes in NK cell phenotype following surgical intervention.

## Materials and methods

### Patient cohorts and sample collection: SMART and RESIST Senior Individuals (SI) cohort

**Fig. 1:**
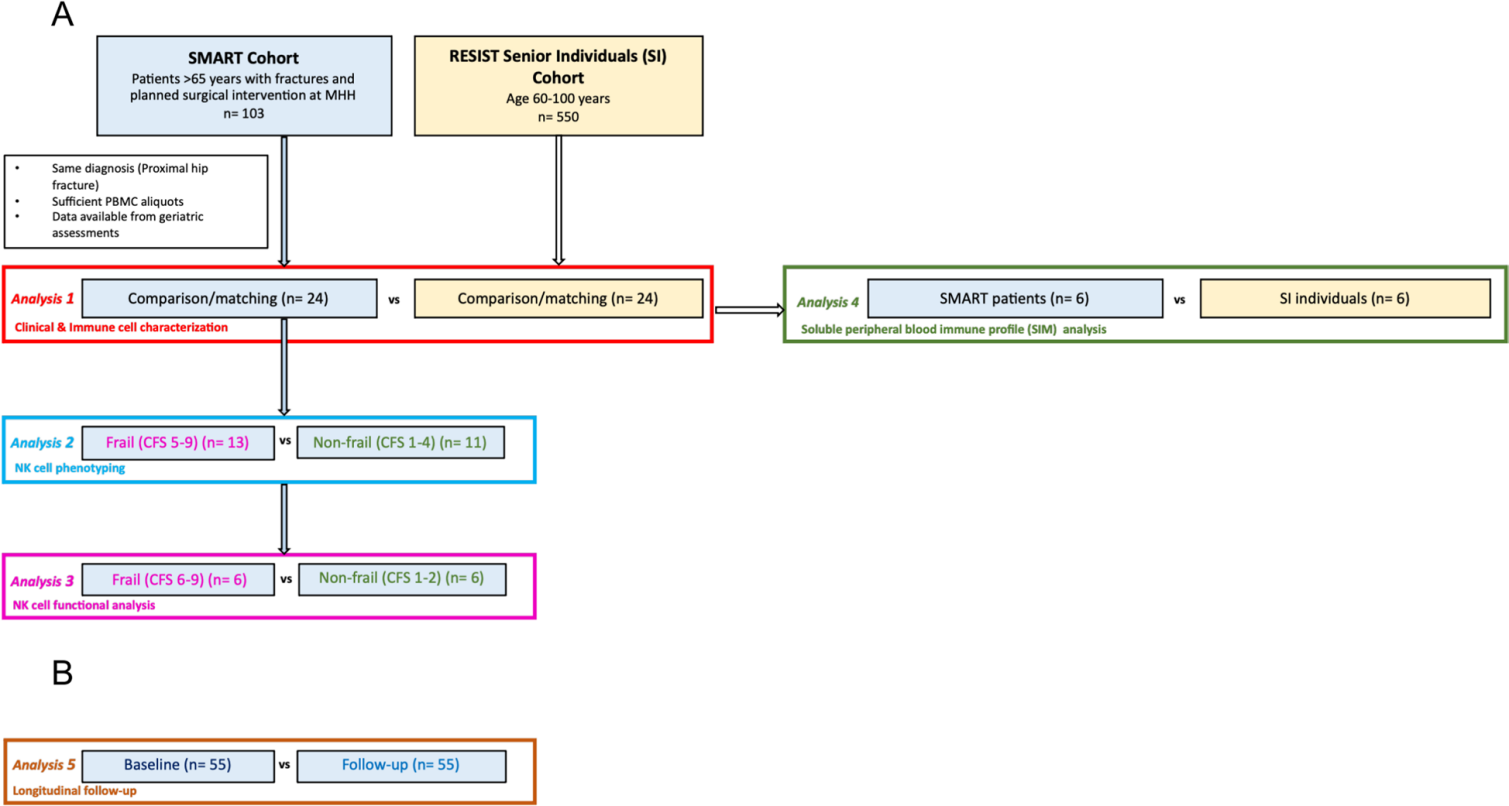
Study design and analysis overview. Flowchart illustrating the structure of the study cohorts and an overview of analyses performed. In both cohorts (SMART and SI), clinical data and biological samples were collected in accordance with geriatric assessments performed. **A**, **B** In the SMART cohort, flow cytometry data were collected prior to surgery and at follow-up (3–6 ± 1 days) after surgery, whereas no follow-up data were available for the SI cohort. The study included multiparametric flow cytometry and immune cell characterization (Analysis 1, **A**), NK cell phenotyping comparing frail and non-frail SMART patients (Analysis 2, **A**), NK cell functional readout in CFS-stratified subgroups (Analysis 3, **A**), soluble immune mediator profiling in SMART patients versus SI individuals (Analysis 4, **A**), and longitudinal NK cell phenotyping before and after surgery in SMART patients (Analysis 5, **B**)

In total, 103 patients aged > 65 years with acute fractures requiring surgical intervention were hospitalized between January 2023 and March 2024 in the Department of Trauma Surgery at Hannover Medical School and recruited within the “SMART Cohort”. Written informed consent was obtained from all participants prior to study inclusion. The study was approved by the Ethics Committee of Hannover Medical School (10963_BO_K_2023). Patients with immunosuppressive medication, chemotherapy or immunotherapy, relevant bacterial or viral infections or anemia < 7 g/dL were excluded. Peripheral blood samples were collected preoperatively during routine clinical care. In addition, postoperative samples were obtained from a subset of patients on day 3 (± 1) and day 6 (± 1) after surgery (Fig. 1). In 50 patients, six comprehensive geriatric assessments were performed: Clinical Frailty Scale (CFS), Geriatric depression scale (GDS), Lawton Scale (IADL), Mini Mental Status Test (MMSE), Barthel Index and Parker Mobility Scale (PMS). Moreover, the patients' grip strength was tested. Demographic and clinical data were recorded, including laboratory parameters, body weight and height.

The RESIST SI cohort (further referred as SI cohort) consists of 650 healthy individuals, 550 of whom are aged 60–100 years and were randomly selected through the residents’ registry of the city of Hannover, the capital of Lower Saxony, Germany [19]. Participation was voluntary, and written informed consent was obtained from all individuals. The study was approved by the Ethics Committee of Hannover Medical School (8615_BO_S_2019). Individuals with a history of organ transplantation were excluded. Detailed cohort characteristics have been published previously [19]. Comprehensive clinical questionnaires including medical history, socio-demographic variables, vaccination and medication status, as well as standardized clinical examinations and biomaterial collection (e.g. peripheral blood), were conducted [19].

For further comparative analyses, participants of the SMART and SI cohorts were matched for age and sex to control for their potential confounding effects. Matching was carried out after exclusion of patients with fractures other than proximal hip fractures, insufficient peripheral blood samples, or missing geriatric assessment data to ensure comparability between groups (Fig. 1A). Nearest-neighbor matching was performed using R software (version 4.3.2) and the MatchIt package [20]. After matching, differences between the cohorts were tested for statistical significance.

### Geriatric assessments

Frailty assessment in both the SMART and SI cohorts included the Barthel Index and hand grip strength measurements (Supplementary Table 1.1). The Barthel Index is an established measure of functional independence in activities of daily living, ranging from 0 (complete dependence) to 100 (full independence) [21]. Hand grip strength was assessed using a Jamar handheld dynamometer for both hands; three measurements were performed per side, and the mean value of the dominant hand was used for analysis [22].

Several additional assessments were conducted only in the SMART cohort (Supplementary Table 1.2). Frailty severity was further evaluated using the Clinical Frailty Scale (CFS; 1–9) [23]. Pre-fracture mobility was assessed using the Parker Mobility Score (PMS), specifically developed for hip fracture patients (range 0–9) [24]. Functional independence in daily instrumental activities was evaluated using the IADL scale across eight domains [25]. Cognitive function was assessed with the Mini-Mental State Examination (MMSE; maximum score 30) [26]. Depressive symptoms were screened using the Geriatric Depression Scale (GDS; 0–15) [27]. In the SI cohort, cognitive performance was assessed using the Montreal Cognitive Assessment (MoCA; maximum 30 points), and depressive symptoms were evaluated using the Beck Depression Inventory (BDI; score range 0–63) [28, 29].

### Sample preparation, isolation and storage of PBMC and plasma

Fresh whole blood was collected in citrate phosphate dextrose adenine (CPDA) monovettes for peripheral blood mononuclear cell (PBMC) isolation and in ethylene diamine tetraacetic acid (EDTA) monovettes for plasma collection. After blood draw, samples were centrifuged at 2000 × g for 10 min, and plasma was aliquoted and stored at −80 °C at Hannover Unified Biobank until further use. PBMC were isolated according to institutional standard operation procedures using Ficoll density-gradient centrifugation, cryopreserved, and stored in liquid nitrogen until further analysis [30].

### Immune cell characterization using flow cytometry measurements

PBMC from the SMART and SI cohorts were analyzed using previously validated multicolor panels [19] (Supplementary Tables 22.1—2.2). The same antibody panels, staining protocols, instrumentation, and gating strategies (Supplementary Fig. 1) were applied to both cohorts to minimize batch effects. A 29-color spectral multiparametric panel (Supplementary Table 2.3) was additionally used for the SMART cohort. For NK cell-focused follow-up analyses, an additional 25-color panel was applied (Supplementary Table 2.4). In more detail, cryopreserved PBMCs were thawed, washed twice with FACS buffer (PBS containing 2% FCS and 2 mM EDTA), and plated at 0.5 × 10⁶ cells per well. Cells were subsequently stained with the appropriate combinations of fluorochrome-conjugated monoclonal antibodies for surface markers. Surface staining was performed for 15 min at room temperature in the dark, followed by intracellular staining for 30 min after fixation and permeabilization using either the eBioscience FOXP3 Staining Buffer Set or BD Cytofix/Cytoperm Fixation/Permeabilization Kit. Dead cells were excluded by staining with Live/Dead Blue (Thermofisher) or Zombie NIR (BioLegend). All antibodies used for staining are listed in Supplementary Tables 2.1–2.5. Samples were acquired on a 4- or 5-laser spectral flow cytometer (Cytek Aurora, Cytek Biosciences, Fremont, California). Single-stained controls were used for unmixing, and compensation was applied during data acquisition. FlowJo software v10.10.0 (BD Biosciences) was used for data analysis.

For high-dimensional analysis, 3,000 events per sample were exported, barcoded, concatenated, and analyzed using Uniform Manifold Approximation and Projection (UMAP v4.1.1) [31] and Phenograph (v4.0.5) clustering [32] via publicly available FlowJo plugins.

### NK cell function after stimulation

Thawed PBMC were seeded in 96-well plates at 0.5 × 10^6^ cells per well and stimulated for 24 h with IL‐12 (10 ng/mL; Miltenyi Biotec, Bergisch Gladbach, Germany), IL-15 (100 ng/mL; Miltenyi Biotec), and IL‐18 (100 ng/mL; MBL International Corporation, Woburn, MA) in human serum AB medium at 37 °C and 5% CO_2_. During the last 6 h of stimulation, Brefeldin A and CD107a antibody were added. For each sample, an unstimulated control was included. After 24 h, surface and intracellular staining was performed as previously described [30], and samples were acquired on a LSR Fortessa flow cytometer (BD Biosciences). Antibodies used for the functional assays are listed in Supplementary Table 2.5.

### Soluble immune marker (SIM) measurement

Plasma levels of 92 soluble immune biomarkers, including cytokines, chemokines and growth factors, were quantified using the Olink Target 96 Inflammation panel (Olink Proteomics, Sweden), which employs a proximity extension assay (PEA) for high-throughput, multiplex protein detection. Plasma samples were processed according to the manufacturer's instructions. Data were reported as normalized protein expression (NPX) values, a log2-transformed relative quantification unit enabling comparison across samples. Normalization was performed using R statistical software (Version 4.3.2).

### Statistical analysis

GraphPad Prism software v8.3.1 and v10.3.0 (GraphPad Software, San Diego, CA, USA) were used for statistical analyses. All datasets were tested for normal distribution using the D’Agostino-Pearson normality test. For comparisons between two unpaired, normally distributed groups, a two-tailed unpaired t-test was used. Non-normally distributed datasets were analyzed using the Mann–Whitney U test. Correlations between datasets were analyzed using Pearson’s r for normally distributed variables or Spearman’s r for non-normally distributed or ordinal variables. Level of significance was defined as *p* < 0.05 (*), *p* < 0.01 (**), *p* < 0.001 (***), *p* < 0.0001 (****).

## Results

### Study design and analysis overview

To study the impact of frailty on the immune system, we established a prospective cohort of older patients hospitalized with acute fractures requiring surgical intervention (SMART cohort). Between January 2023 and March 2024, 103 patients aged ≥ 65 years were recruited. The RESIST Senior Individuals (SI) cohort, an arm of which consists of 550 generally healthy adults aged 60–100 years, served as a control population (Fig. 1A). Subsequently, SMART patients with a diagnosis of proximal femur fracture, complete laboratory data, available geriatric assessment, and sufficient PBMC samples were selected (Fig. 1A). 24 SMART patients met these criteria and were matched by age and sex to 24 SI individuals. This matched dataset formed the basis for comparing baseline characteristics, immune cell composition, and NK cell phenotypes (Analysis 1, Fig. 1A, Supplementary Table 1.1). Within the SMART cohort, patients were stratified into “non-frail” (CFS 1–4; very fit to vulnerable) and frail (CFS 5–9; mildly frail to terminally ill) subgroups for phenotypic analyses (Fig. 1A). NK cell phenotype (Analysis 2) and function (Analysis 3) were evaluated in these groups. Additionally, the soluble immune profile in the peripheral blood was compared between SMART patients and SI individuals (Analysis 4, Fig. 1A). Moreover, NK cell phenotypes were assessed longitudinally before and after surgery in 55 SMART patients to evaluate postoperative NK cell alterations over time (Analysis 5, Fig. 1B).

### Patient baseline characteristics of SMART patients and SI individuals

Baseline characteristics and laboratory parameters of the SMART cohort (n = 103) and the SI cohort (n = 550) are summarized in Table 1. Briefly, patients recruited within the SMART cohort were older and had lower body weight, hemoglobin, and total protein levels, while C-reactive protein (CRP) and leukocyte counts were significantly increased (Table 1). Frailty-associated measures, including Barthel Index and grip strength, were also markedly reduced in the SMART cohort, consistent with diminished functional status. No statistically significant differences were observed in kidney or liver function between both cohorts (Table 1). LDL cholesterol, total cholesterol, total protein and uric acid levels were significantly lower in SMART patients than in SI individuals (Table 1). In the matched analysis of 24 SMART patients and 24 SI individuals, reduced grip strength, Barthel Index, and total protein concentrations remained significantly lower in SMART patients compared with matched SI individuals (Fig. 1A, Analysis 1, Supplementary Table 1.1). Likewise, CRP and leukocyte counts remained significantly elevated, indicating a heightened inflammatory state in SMART patients (Supplementary Table 1.1).

**Table 1:**
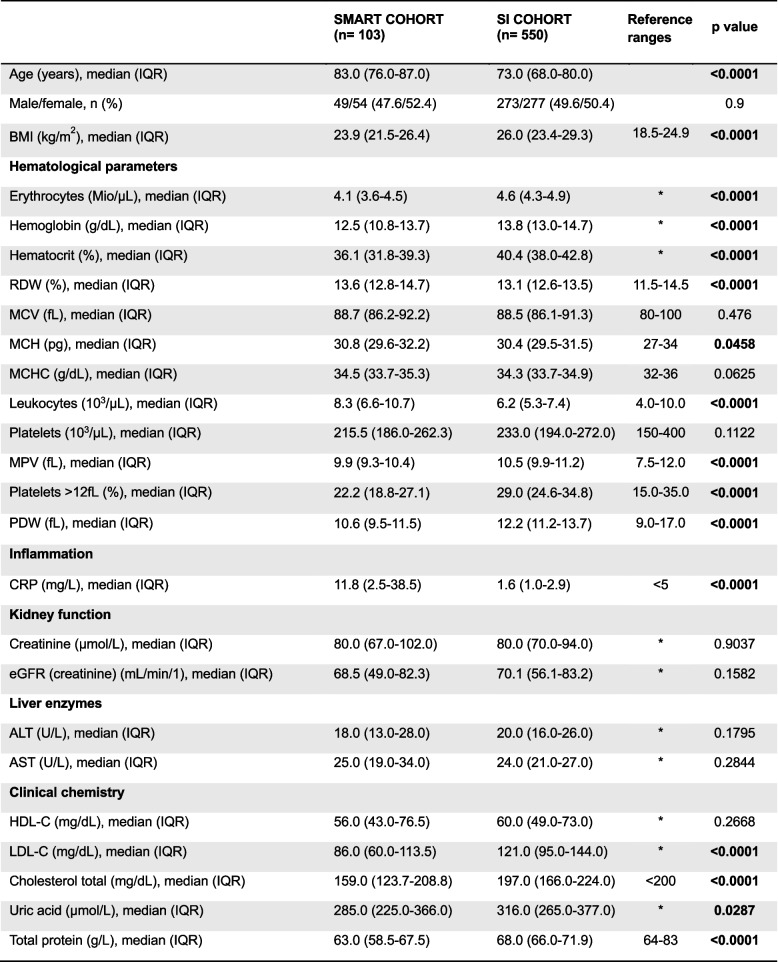
Patient baseline characteristics of SMART patients and SI individuals

Continuous variables are presented as median values with interquartile ranges (IQR), and categorical variables as absolute numbers with percentages. Group comparisons were performed using the Mann-Whitney U test for non-normally distributed data and unpaired t-tests for normally distributed data. Level of significance was defined as p<0.05

Reference ranges are based on standard adult clinical laboratory reference intervals but may vary between laboratories and analytical methods

*ALT *alanine aminotransferase, *AST *aspartate aminotransferase, *BMI *body mass index, *CRP *C-reactive protein, *eGFR *estimated glomerular filtration rate, *HDL-C *high-density lipoprotein cholesterol, *LDL-C *low-density lipoprotein cholesterol, *MCH *mean corpuscular hemoglobin, *MCHC *mean corpuscular hemoglobin concentration, *MCV *mean corpuscular volume, *MPV *mean platelet volume, *PDW *platelet distribution width, *RDW *red cell distribution width

* Reference ranges are sex dependent

Taken together, SMART patients were frailer, reflected by reduced grip strength and lower Barthel scores, and displayed an inflammatory state, as indicated by elevated CRP levels and leukocyte counts compared with matched SI individuals.

### Geriatric characterization of SMART patients and SI individuals

To characterize the SMART cohort in the context of frailty, comprehensive geriatric assessments were performed (Supplementary Table 1.2). SMART patients showed a median CFS score of 5, indicating a moderate degree of frailty (Supplementary Table 1.2). Mobility assessment using the PMS yielded a median score of 6 (Supplementary Table 1.2), indicating that the participants were moderately mobile or required at least some support. Cognitive function, evaluated with the MMSE, showed a median score of 26 (Supplementary Table 1.2), determining the presence of mild cognitive impairment. Functional independence assessed by the IADL scale had a median score of 6 (Supplementary Table 1.2), indicating mild difficulties with independent living and in performing some daily activities. To screen for late-life depression, the GDS was applied, resulting in a median score of 3 (Supplementary Table 1.2), which falls within the range indicating no or only minimal depressive symptoms. Although identical assessments were not conducted in the SI cohort, tests for depression and cognitive function were similarly performed. The MoCA result yielded a median score of 24, indicating no cognitive dysfunction, while the BDI median score of 3 suggested no or only minimal depressive symptoms (Supplementary Table 1.3).

In summary, these results show that SMART patients exhibited a moderate level of frailty and mild cognitive impairment, which together validates our cohort for subsequent downstream analysis.

### NK cells are diminished in SMART patients and correlate with grip strength

**Fig. 2:**
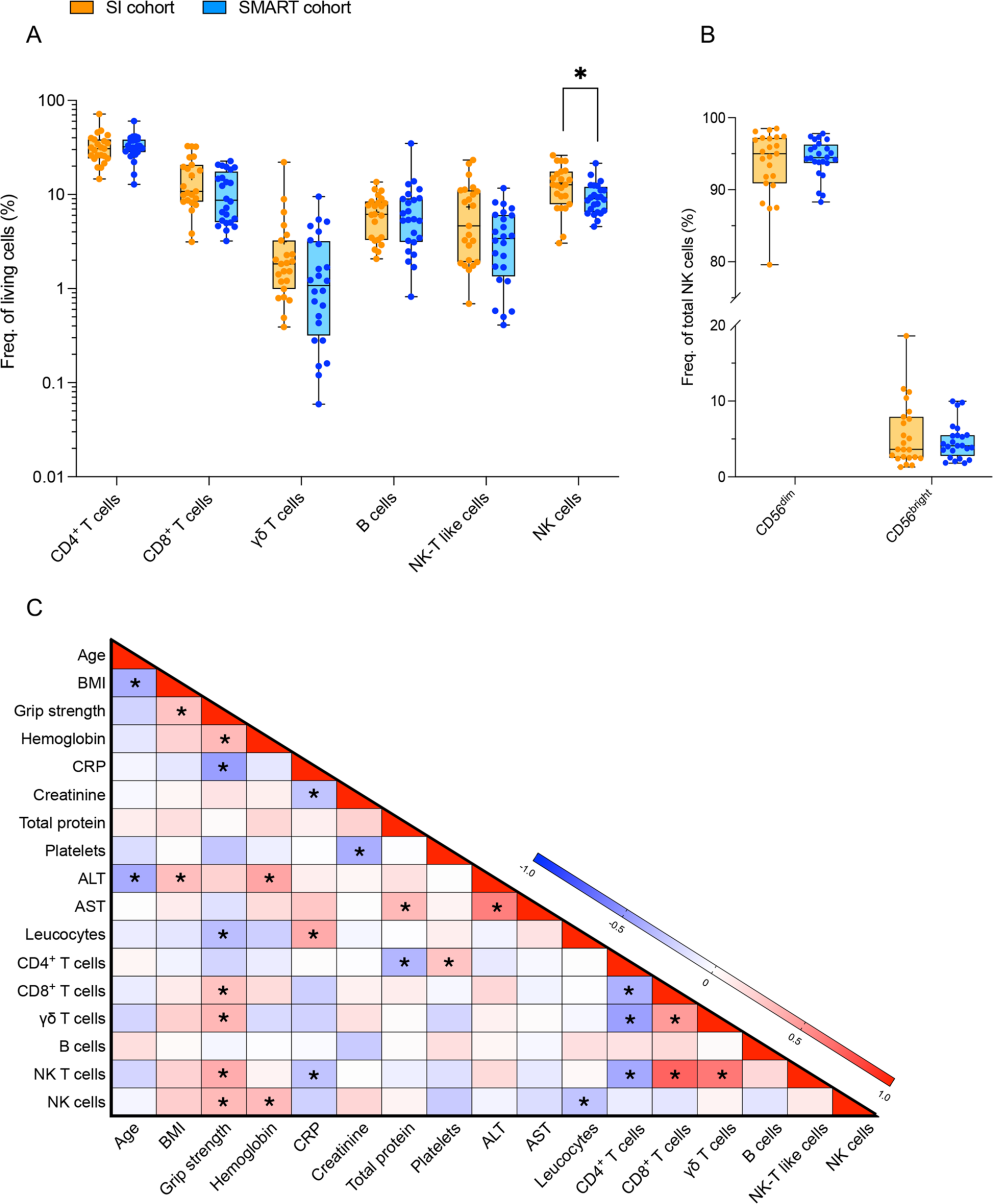
NK cells are diminished in SMART patients and correlate with frailty. **A** Frequencies of indicated effector cell subsets in SMART patients (n = 24) compared with SI individuals (n = 24). **B** Frequencies of CD56^dim^ and CD56^bright^ NK cells in SMART patients (n = 24) versus SI individuals (n = 24). Bars represent mean values and error bars indicate standard deviation (SD) of the mean. Data were tested for normal distribution, and either an unpaired t-test or a Mann–Whitney U test was applied as appropriate. **C** Correlation matrix of clinical markers, laboratory values, and effector cell frequencies from both cohorts combined (n = 48). Spearman’s rank correlation coefficients (ρ) are shown, with positive (red) and negative (blue) correlations scaled by color intensity. Level of significance was defined as *p* < 0.05 (*). ALT, alanine aminotransferase; AST, aspartate aminotransferase; CRP, C-reactive protein. Sex distribution male/female: 9/15 in each cohort

To assess whether frailty is associated with alterations in immune cell composition, we performed multiparametric flow cytometry in SMART patients and matched SI individuals (Fig. 2A). Among all analyzed effector cell populations, only the NK cell frequencies were significantly reduced in SMART patients (Fig. 2A). The proportions of CD56^dim^ and CD56^bright^ NK cell subsets remained unchanged between the two cohorts (Fig. 2B). Next, we evaluated whether frequencies of effector cell populations, particularly NK cells, were associated with sex (Supplementary Fig. 2), clinical measures of physical function and inflammation (CRP and leukocyte counts) (Fig. 2C). Grip strength, a widely used measure of physical function and frailty, showed a significant positive correlation with NK cell frequencies (Fig. 2C). Moreover, BMI and hemoglobin were also significantly associated with grip strength, whereas CRP and leukocyte counts correlated inversely, indicating that reduced physical performance was linked to heightened systemic inflammation (Fig. 2C). To address potential confounding by cohort differences, we additionally assessed these relationships within the SMART cohort. While no significant association between NK cell frequencies and frailty status was observed, the positive relationship between NK cell frequencies and grip strength remained evident (Supplementary Fig. 3).

Taken together, NK cell frequencies are reduced in SMART patients compared with SI individuals. Increased CRP levels and leukocyte counts were associated with poorer physical performance in both the combined cohort analysis and the analysis of the SMART cohort alone. However, no significant association with frailty status was observed within the SMART cohort.

### NK cells in SMART patients display a distinct phenotype

**Fig. 3:**
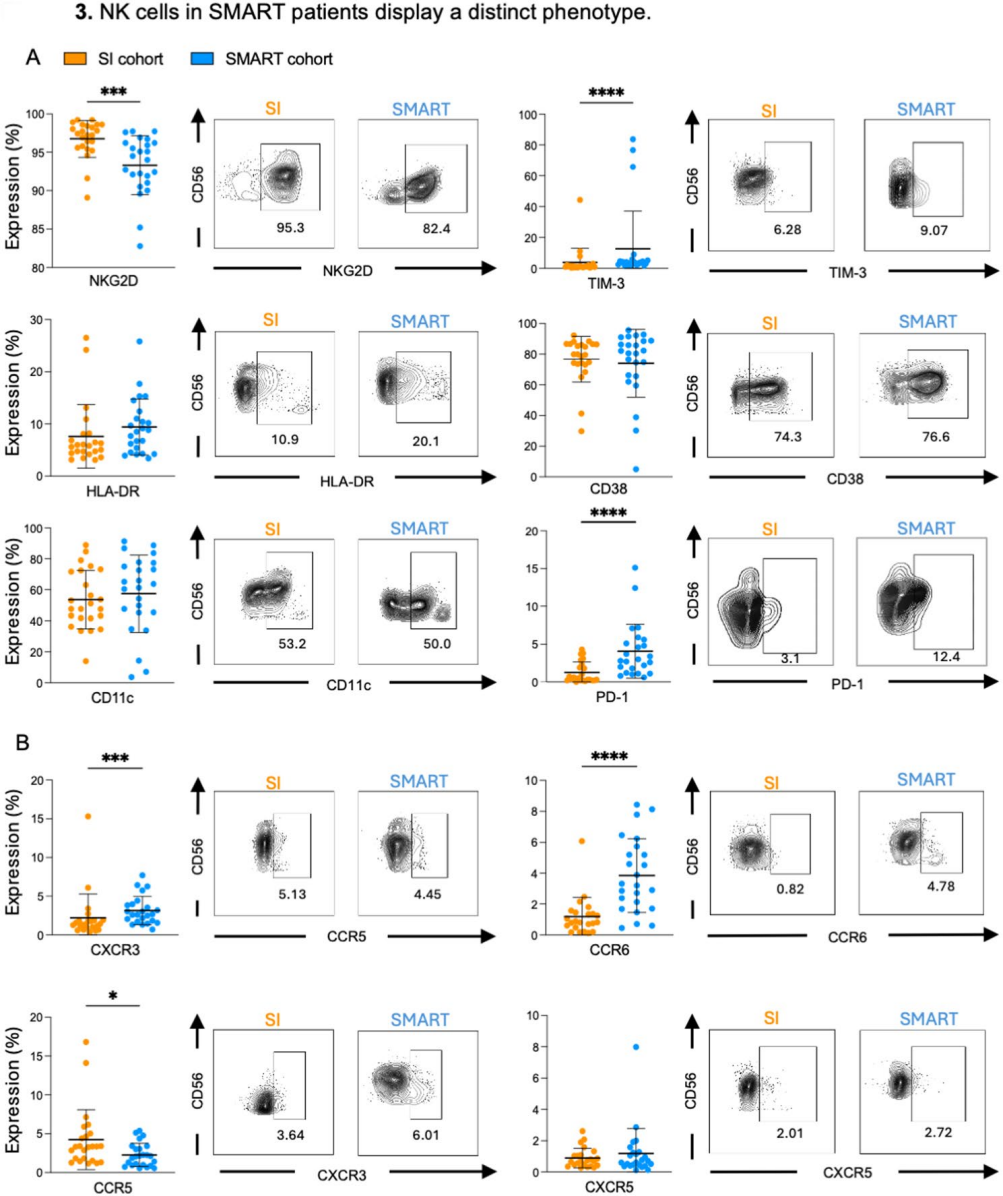
NK cells in SMART patients show a distinct phenotype. **A** Expression of activation/exhaustion markers, **B** and chemokine receptors on total NK cells from SMART patients (n = 24) compared with SI individuals (n = 24). Representative flow cytometry plots for each marker are displayed next to the data graphs. Bars represent mean values and error bars indicate standard deviation (SD). Data were assessed for normality and analyzed using unpaired t-tests or Mann–Whitney U tests, as appropriate. *p* < 0.05 (*), *p* < 0.01 (**), *p* < 0.001 (***), *p* < 0.0001 (****). Sex distribution male/female: 9/15 in each cohort

As we observed a reduction of NK cell frequencies in SMART patients compared with individuals of the SI cohort, we next examined the NK cell phenotype in more detail (Fig. 3). NK cells from SMART patients displayed significantly lower expression of NKG2D and higher expression of PD-1 and TIM-3 compared with SI individuals (Fig. 3A). In contrast, no significant differences were observed for HLA-DR, CD38 and CD11c expression (Fig. 3A). Assessment of chemokine receptors revealed reduced CCR5 expression on NK cells from SMART patients, whereas CCR6 and CXCR3 were significantly increased compared with SI individuals (Fig. 3B). In addition, similar observations were detectable in CD56^dim^ and CD56^bright^ NK cell subsets (Supplementary Fig. 4).

In summary, NK cells in SMART patients exhibit a distinct phenotypic profile characterized by altered expression of markers linked to activation, cytotoxic activity, inhibitory signaling, and chemokine receptor expression, as compared to SI individuals.

### High-dimensional analysis of NK cells in frail compared with non-frail patients within the SMART cohort

**Fig. 4:**
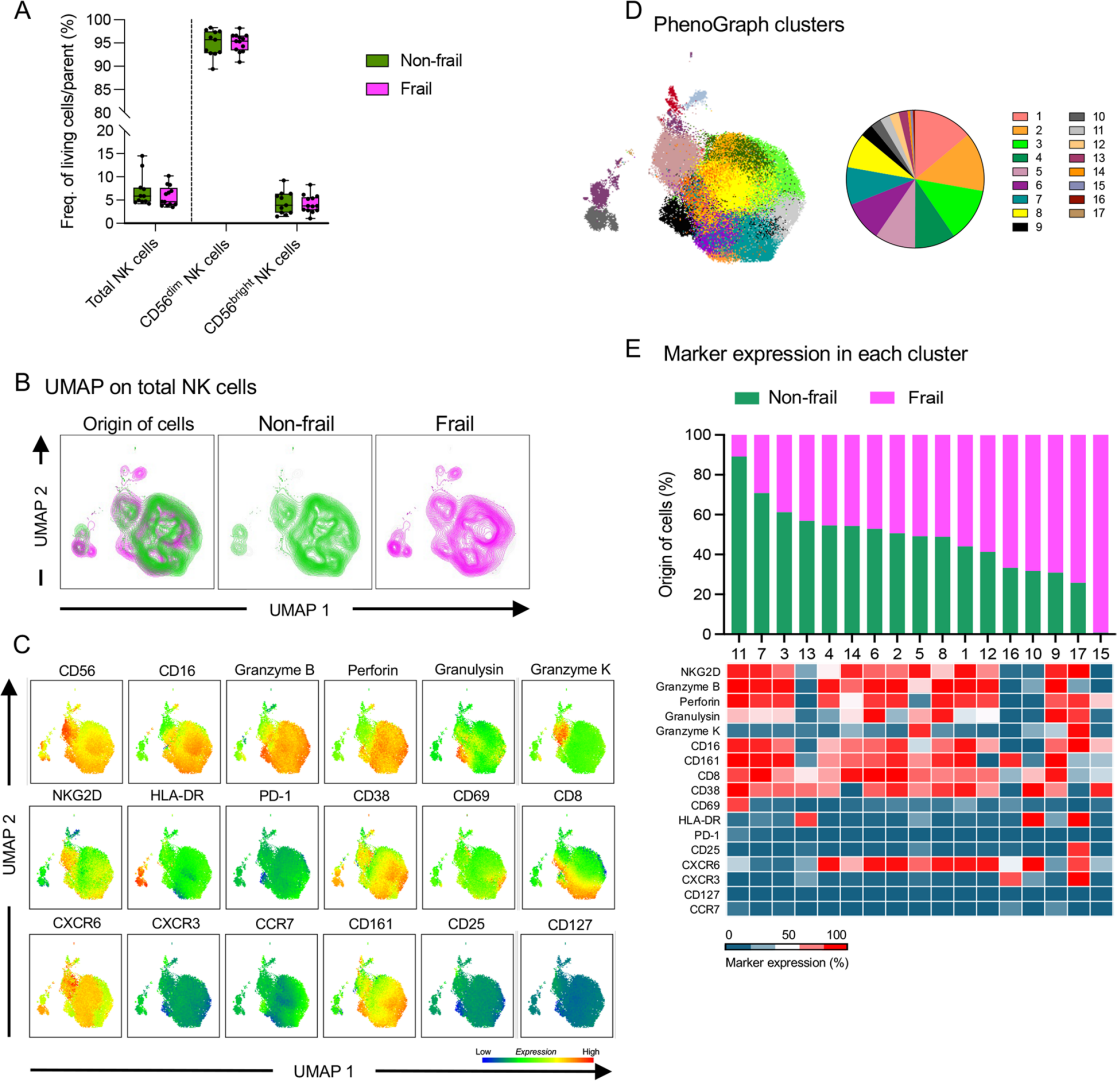
High-dimensional analysis of NK cells in frail compared with non-frail patients within the SMART cohort. **A** Frequencies of total NK cells, CD56^dim^, and CD56^bright^ subsets in frail (n = 13) compared to non-frail (n = 11) patients. Bars indicate mean values, and error bars represent standard deviation (SD) of the mean. **B** UMAP visualization of total NK cells, clustering based on surface marker expression, stratified by frailty status. **C** Expression levels of each indicated marker in the UMAP analysis. Per group 13 frail and 11 non-frail patients with each 3000 NK cells were exported, concatenated and analyzed using the publicly available FlowJo **B**, **C** UMAP and **D** PhenoGraph plugins (FlowJo, BD Biosciences). **D** Pie chart indicates the proportion of the identified Phenograph clusters from total concatenated NK cells. **E** Heatmap displaying indicated marker expression profiles across PhenoGraph-defined clusters, and the relative contribution of frail and non-frail NK cells to each cluster. Data were tested for normal distribution, and either unpaired t-tests or unpaired Mann–Whitney U tests were performed when indicated. Sex distribution male/female: 9/15 in each cohort

Based on these findings, we next characterized NK cell heterogeneity within the SMART cohort in more depth. Therefore, SMART patients were stratified into frail (CFS 5–9) and non-frail (CFS 1–4) patients according to their CFS scores (Fig. 1A). Multiparametric flow cytometry was performed using a distinct panel to assess NK cell activation, exhaustion and cytotoxicity (Supplementary Table 2.3). NK cell frequencies and the distribution of CD56^dim^ and CD56^bright^ subsets did not differ significantly between frail and non-frail patients (Fig. 4A). Next, we applied high-dimensional UMAP analysis followed by PhenoGraph clustering on total NK cells from frail and non-frail patients (Fig. 4B-E). Of note, NK cells from frail and non-frail patients displayed a substantial overlap (Fig. 4B). Nevertheless, individual marker mapping showed minor differences in activation- and tissue-homing-associated features (Fig. 4C). In more detail, frail patients exhibited higher expression of HLA-DR and CD38 and lower expression of CXCR6 and granzyme B across the NK cell compartment (Fig. 4C). In contrast, conventional analysis revealed lower PD-1 expression in frail patients within both CD56^dim^ and CD56^bright^ NK cell subsets. Notably, this finding was restricted to the subset analyses and was not observed at the level of total NK cells (Supplementary Fig. 5). Cluster-level analysis identified no uniform shift between frail and non-frail NK cells; rather, differences were confined to specific subsets (Fig. 4D, E). Clusters enriched in non-frail patients displayed higher expression of NKG2D and granzyme B, whereas clusters enriched in frail individuals were characterized by increased granzyme K expression (Fig. 4D, E).

Taken together, these exploratory analyses revealed only modest cluster differences between frail and non-frail SMART patients. These differences were substantially smaller than those observed between SMART patients and SI individuals, suggesting a limited impact of frailty status on the NK cell phenotypes assessed in this study. This implicates that the immunological shifts in NK cells are driven primarily by the combined effects of aging, frailty, acute fracture, surgery, and hospitalization rather than by frailty status alone.

### NK cell response to cytokine stimulation in frail compared with non-frail patients within the SMART cohort

**Fig. 5:**
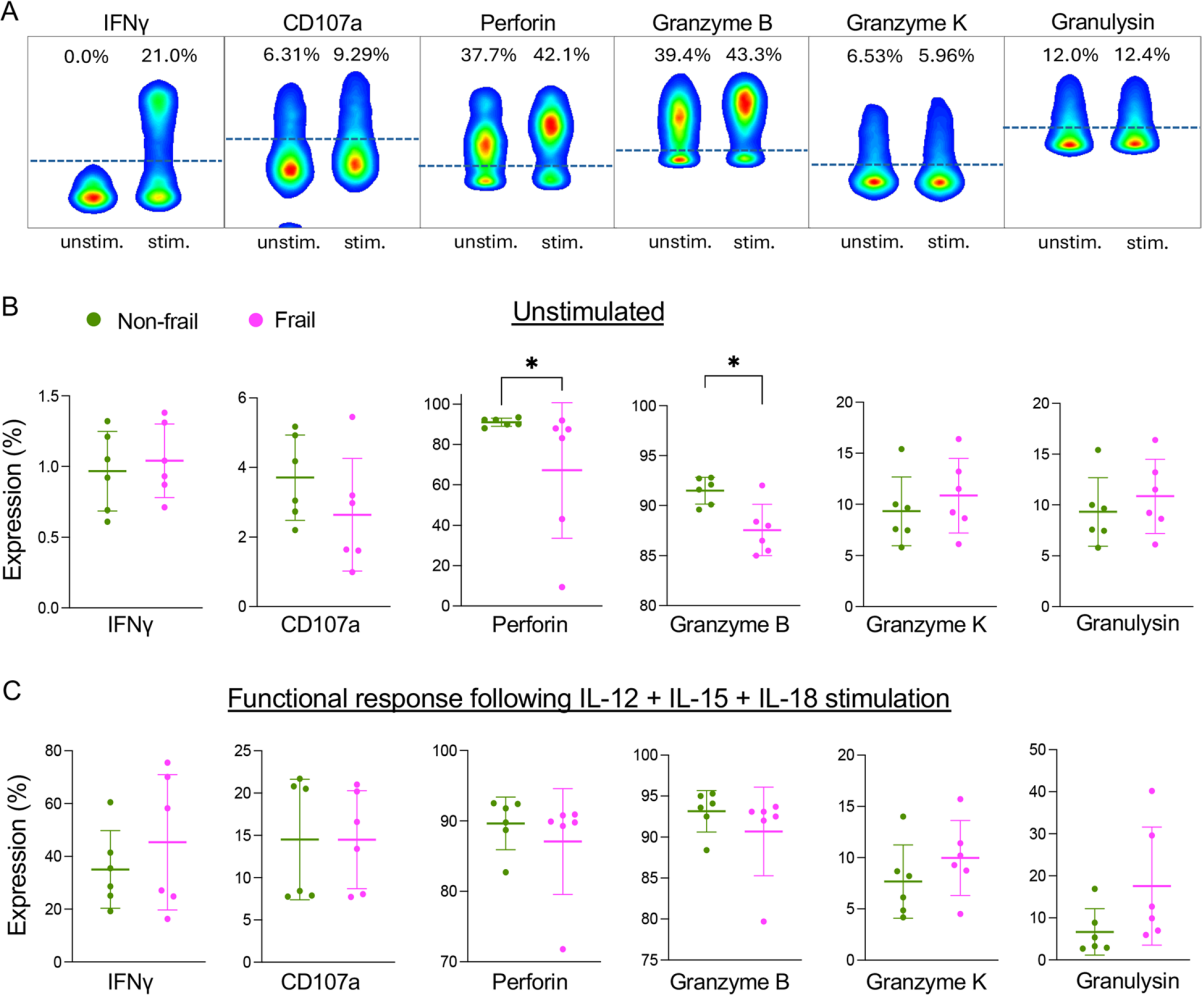
NK cell response to cytokine stimulation in frail compared with non-frail patients within the SMART cohort. NK cells from SMART patients (n = 12) were assessed for cytokine-induced functional responses. Six non-frail patients (CFS 1–2) and six frail (CFS 6–9) patients were selected and compared with each other. PBMCs were stimulated with IL-12 + IL-15 + IL-18 or left untreated. **A** Representative flow cytometry plots of functional responses in total NK cells upon cytokine stimulation. **B** Frequencies of indicated proinflammatory cytokines and effector molecules in unstimulated NK cells from frail and non-frail patients. **C** Functional responses following IL-12 + IL-15 + IL-18 stimulation comparing frail and non-frail patients. Bars indicate mean values, and error bars represent standard deviation (SD). Data were tested for normal distribution, and either an unpaired t-test or a Mann–Whitney U test was applied as appropriate. *p* < 0.05 (*). Sex distribution male/female: 3/9

To assess NK cell responsiveness to cytokine stimulation, we selected six patients with the lowest CFS scores (CFS 1–2) and six matched patients with the highest CFS scores (CFS 6–9) from the SMART cohort. PBMC were stimulated with the innate-like cytokines IL-12, IL-15, and IL-18, or left untreated (Fig. 5A). Indeed, cytokine stimulation induced robust upregulation of IFNγ and CD107a in both groups (Fig. 5A, Supplementary Fig. 6). Furthermore, without stimulation, significantly lower expression of the cytotoxicity-associated molecules perforin and granzyme B were observed in frail patients compared with non-frail patients (Fig. 5B). Despite minor trends towards higher IFNγ, granzyme K, and granulysin production in frail patients, NK cells showed largely comparable functional responses upon stimulation (Fig. 5C).

In summary, frail patients exhibited lower baseline expression of perforin and granzyme B, whereas cytokine-induced NK cell responses were largely preserved.

### A proinflammatory cytokine milieu in SMART patients is associated with reduced NK cell frequency

**Fig. 6:**
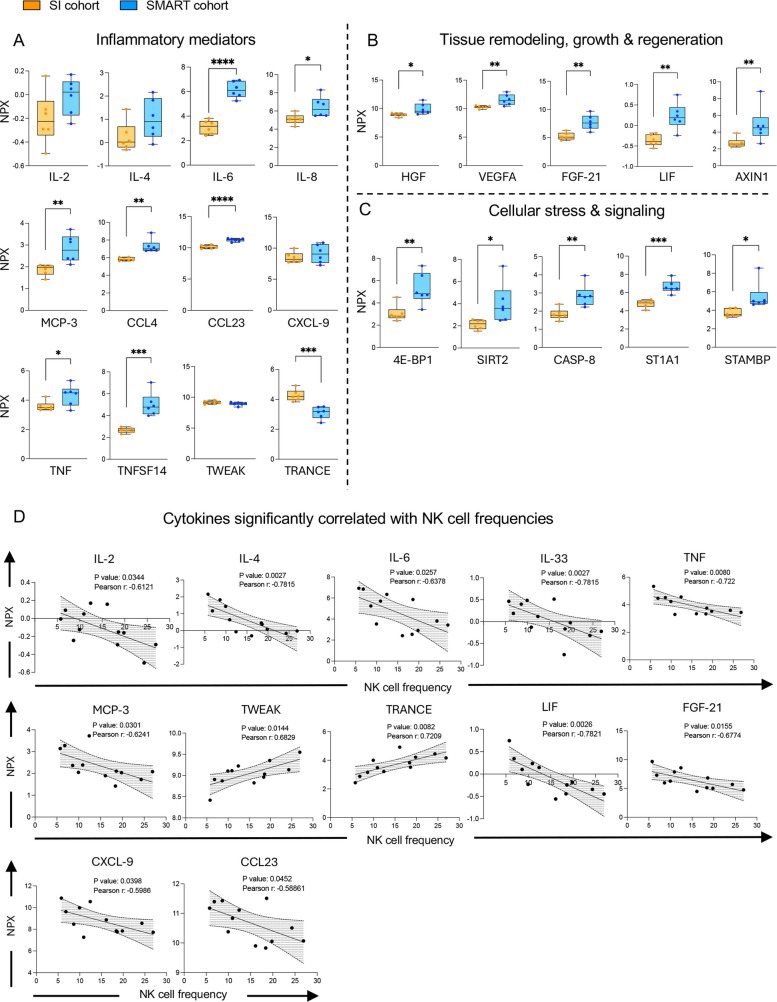
SMART patients exhibit a distinct cytokine milieu that is associated with reduced NK cell frequencies. **A** Plasma levels of proinflammatory cytokines, **B** tissue remodeling and regeneration factors, and **C** cellular stress or signaling molecules were compared between SMART patients (n = 6) and SI individuals (n = 6). Soluble immune mediators were quantified using the Olink Target 96 Inflammation panel, and normalized protein expression (NPX) values are shown. Bars indicate mean values, and error bars represent standard deviation (SD) of the mean. Data were tested for normal distribution, and subsequently either an unpaired t-test or a Mann–Whitney U test was applied as appropriate. **D** Cytokines significantly correlated with NK cell frequencies across all participants from both cohorts (n = 12). Regression lines with 95% confidence bands and corresponding correlation coefficients are displayed. Correlations were performed using Pearson’s or Spearman’s coefficients depending on data distribution. *p* < 0.05 (*), *p* < 0.01 (**), *p* < 0.001 (***), *p* < 0.0001 (****). NPX, normalized protein expression. Sex distribution male/female: 6/6 in each cohort

To better characterize the systemic immune environment associated with frailty and acute fracture, soluble immune mediators (SIMs) were quantified in SMART patients (n = 6) and matched SI individuals (n = 6) (Fig. 6A-C). This analysis was restricted to individuals with complete cytokine measurements, corresponding NK cell analyses, and comprehensive geriatric assessments, all from patients with the same diagnosis to ensure dataset consistency.

Compared to the SI cohort, SMART patients displayed a distinct proinflammatory and immunomodulatory plasma profile. In more detail, levels of inflammatory cytokines (IL-6, IL-8, TNF, TNFSF14) and chemokines (MCP-3, CCL4, CCL23) were significantly elevated in SMART patients, indicating enhanced systemic immune activation and leukocyte recruitment (Fig. 6A). Additionally, several growth and tissue repair factors (HGF, VEGFA, FGF-21, LIF) were also significantly increased (Fig. 6B). Furthermore, markers involved in cellular stress responses and immune regulation, including AXIN1, 4E-BP1, SIRT2, CASP-8, ST1A1, and STAMBP were significantly elevated in SMART patients compared with SI individuals (Fig. 6C). Notably, TRANCE (also known as RANKL) was the only analyte, that was reduced in the SMART cohort (Fig. 6A). In addition, no sex-specific differences in SIM were observed (Supplementary Fig. 7).

To explore potential relationships between systemic immune factors and NK cell homeostasis, we performed correlation analyses between plasma SIM concentrations (NPX values) and NK cell frequencies (Fig. 6D). Indeed, significantly negative correlations between NK cell frequencies and several proinflammatory SIMs, including IL-2, IL-4, IL-6, IL-33, TNF, MCP-3, LIF, FGF21, CXCL9, and CCL23 were observed, whereas TWEAK and TRANCE correlated positively with NK cell frequencies. (Fig. 6D).

Overall, SMART patients exhibit a distinct proinflammatory cytokine milieu compared with SI individuals, which is associated with reduced circulating NK cell frequencies.

### Longitudinal NK cell analysis indicates postoperative phenotypic changes

**Fig. 7:**
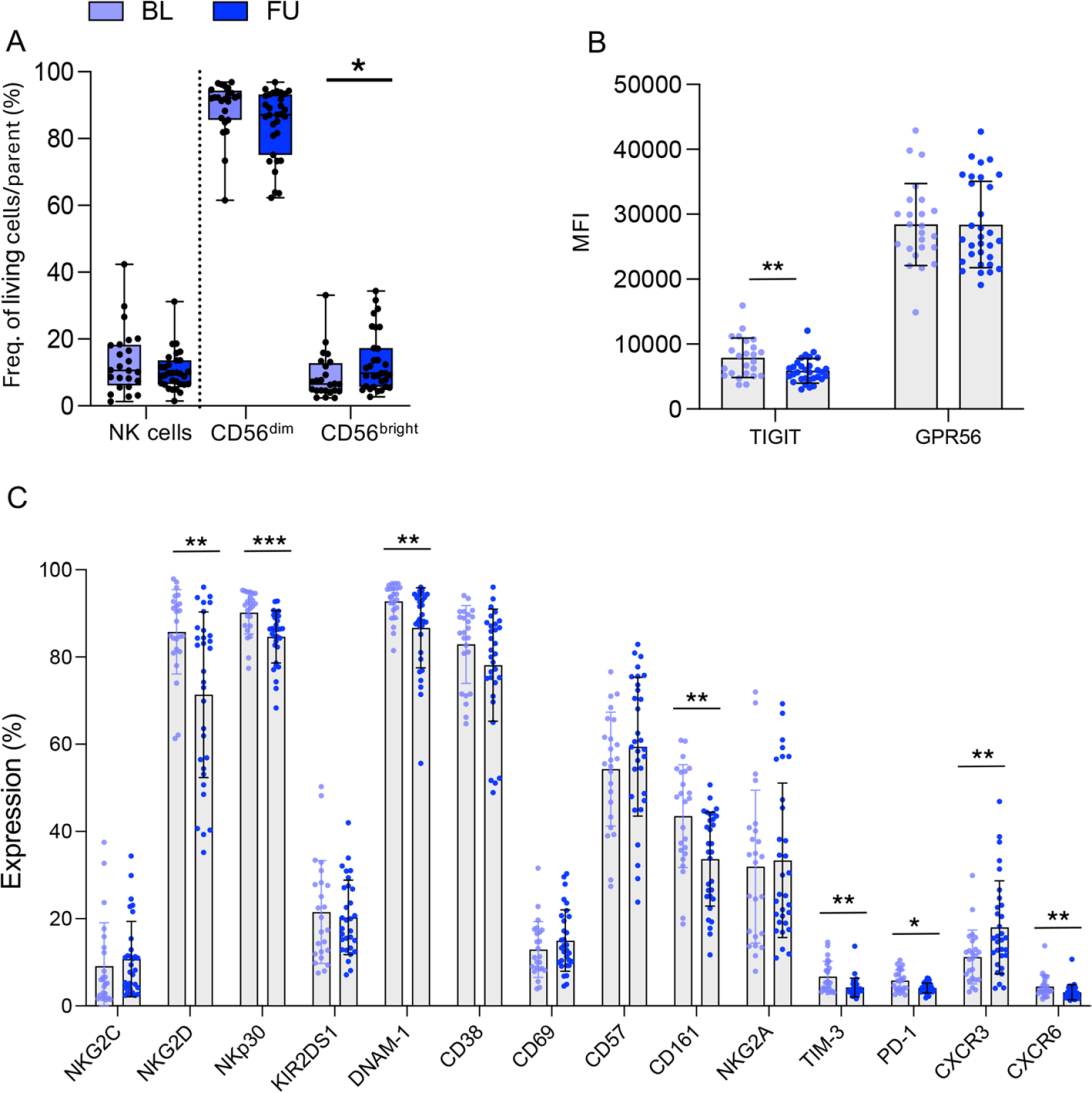
Longitudinal NK cell analysis indicates postoperative phenotypic changes. **A** Frequencies of total NK cells, CD56^dim^, and CD56.^bright^ subsets in SMART patients at baseline (BL, pre-surgery, n = 24) compared to follow-up (FU, 3–6 ± 1 days post-surgery, n = 31). **B** Mean fluorescence intensity (MFI) of indicated phenotypic NK cell markers in SMART patients at BL (n = 24) compared with FU (n = 31). **C** Expression of activation/differentiation, inhibition/exhaustion, and homing markers on total NK cells from SMART patients at BL (n = 24) compared with FU (n = 31). Bars indicate mean values, and error bars represent standard deviation (SD) of the mean. Data were tested for normal distribution, and subsequently either an unpaired t-test or a Mann–Whitney U test was applied as appropriate. *p* < 0.05 (*), *p* < 0.01 (**), *p* < 0.001 (***). BL, baseline; FU, follow-up; MFI, mean fluorescence intensity. Sex distribution male/female: 26/29

Surgical stress and tissue injury are known to modulate immune cell phenotypes [33]. Having shown that NK cells from patients with acute fracture and hospitalization (SMART cohort) exhibit increased PD-1/TIM-3 expression and altered chemokine receptor expression, we next investigated whether these phenotypic differences persist or are further modulated following surgical intervention. To address this, pre-operative baseline (BL) and post-operative follow-up (FU) samples collected on day 3 (± 1) or day 6 (± 1) days after surgery were analyzed longitudinally (Fig. 7A-C). Total NK cell frequencies were comparable between baseline and follow-up, whereas a shift towards CD56^bright^ NK cells was observed after surgery (Fig. 7A). Moreover, several phenotypic changes were observed in the longitudinal analysis (Fig. 7B, C). In more detail NKG2D, NKp30, DNAM-1, TIM-3, PD-1 and TIGIT were significantly downregulated on NK cells at follow-up compared with baseline (Fig. 7B, C). Moreover, expression of the homing-associated receptor CXCR3 was significantly increased on NK cells at follow-up, whereas CXCR6 expression was significantly decreased (Fig. 7C).

To summarize, while overall NK cell frequencies remained stable, surgery was associated with distinct changes in NK cell subset distribution and marker expression during early postoperative follow-up, underscoring the impact of surgical intervention on NK cell phenotypes.

## Discussion

In this study, we demonstrate that older patients hospitalized with acute fractures (SMART cohort) exhibit pronounced alterations in NK cell homeostasis compared with healthy senior individuals (SI cohort). SMART patients showed reduced NK cell frequencies, altered expression of inhibitory and chemokine receptors, and a distinct proinflammatory cytokine milieu. Importantly, these alterations were substantially more pronounced between SMART patients and SI individuals than between frail and non-frail patients within the SMART cohort, underscoring the dominant impact of acute fracture and hospitalization on NK cell biology. These findings indicate that immune vulnerability in this setting is largely context-dependent and cannot be explained by baseline frailty alone.

Clinically, SMART patients displayed features of physical decline, including reduced grip strength, lower BMI, and diminished Barthel Index scores, together with a heightened inflammatory state, indicated by elevated CRP levels and leukocyte counts. These differences persisted after age- and sex-matching and are consistent with previous reports linking frailty to anemia, dyslipidemia, hypoalbuminemia, and systemic inflammation [34–36]. Such alterations likely reflect a catabolic metabolic state and reduced physiological reserve associated with chronic low-grade inflammation [37–39]. Previous studies have suggested that psychological stress and depressive symptoms may contribute to impaired NK cell function following hip fracture [40]. In the present cohort, however, no significant association between depressive symptom scores and NK cell frequencies was observed. Nevertheless, also other factors such as sex, medication and perioperative stress responses may impact NK cell function. Here, analysis of sex-specific differences in effector cell frequencies and cytokine expression revealed no significant differences between male and female participants. However, these findings should be interpreted with caution due to the limited sample size of the subgroup analyses.

Notably, grip strength, a widely used measure of physical function and frailty, correlated positively with NK cell frequencies. In contrast, no significant association between NK cell frequencies and frailty status was observed within the SMART cohort. Together, these findings suggest that NK cell frequencies may be more closely associated with physical function than with frailty status alone. Among all effector cell subsets analyzed, NK cells were uniquely reduced in SMART patients compared with SI individuals. This finding contrasts with studies reporting increased circulating NK cell frequencies with advancing age [12, 41–44], and highlights the importance of distinguishing chronological aging from frailty and acute physiological stress. While age-related NK cell expansion is typically described in healthy older adults, our data indicate that acute fracture and hospitalization are associated with lower peripheral NK cell frequencies. The reduction in circulating NK cell frequencies may reflect changes in NK cell distribution. However, the underlying mechanisms remain unclear. The concomitant alterations in chemokine receptor expression and inflammatory mediator levels raise the possibility that inflammatory signals contribute to the observed NK cell changes. However, the present study does not allow causal conclusions regarding the mechanisms underlying these observations. Phenotypic analyses revealed that NK cells from SMART patients exhibited an altered expression of markers associated with activation and inhibition. Reduced NKG2D and perforin expression together with increased TIM-3 expression distinguished SMART patients from healthy controls [16, 45–48]. However, TIM-3 expression levels were generally low in both cohorts and considerable inter-individual variability was observed. Concurrent alterations in chemokine receptor expression, including CXCR3 and CCR6, suggest changes in pathways involved in NK cell migration and trafficking; but whether these changes are due to altered cell distribution, the state of activation, or other processes remains to be investigated more in detail [13, 49–52]. Together, these findings could support a shift toward an activated and functionally constrained, tissue-directed NK cell phenotype. Such a phenotype may compromise early antimicrobial defense while simultaneously promoting immune cell accumulation at sites of tissue injury. This interpretation is reinforced by the systemic cytokine milieu observed in SMART patients. Elevated levels of IL-6, TNF, TNFSF14, MCP-3, CCL4, and CCL23 indicate a proinflammatory environment in SMART patients, associated with both frailty and acute tissue injury [53–56]. In parallel, increased concentrations of growth and repair factors including HGF, VEGFA, FGF-21, and LIF suggest ongoing tissue remodeling following fracture [57–59]. Several stress- and signaling-related molecules elevated in SMART patients have also been reported in settings of persistent immune activation, including treated HIV infection [60]. This overlap suggests that frailty in combination with acute tissue injury may trigger systemic stress responses that resemble those seen in chronic viral infections. Reduced circulating TRANCE (RANKL) levels in SMART patients may reflect enhanced recruitment to fracture sites and are consistent with studies linking low serum RANKL levels to increased fracture risk [61, 62]. Overall, this cytokine pattern coincided with a potentially proinflammatory and remodeling-oriented environment in fracture patients. The fracture context may help explain both the reduced NK cell frequencies in the circulation observed in SMART patients and their altered chemokine receptor expression.

Correlation analyses further revealed strong inverse associations between NK cell frequencies and multiple proinflammatory cytokines, including IL-2, IL-6, IL-33, TNF, and MCP-3. These cytokines are key mediators of chronic inflammation and have been implicated in age-related immune dysregulation and frailty [63–65]. The observed relationships likely reflect cytokine-driven recruitment of NK cells to injured tissues or broader systemic alterations limiting NK cell retention in the circulation. In contrast, positive correlations with TWEAK and TRANCE suggest that specific regulatory pathways may contribute to maintaining NK cell homeostasis under inflammatory conditions. Taken together, these findings support a model in which inflammatory signaling during acute injury influences innate immune homeostasis. High-dimensional and longitudinal analyses further emphasize the dominant role of acute injury and surgical intervention. Differences between frail and non-frail patients within the SMART cohort were modest, whereas comparisons between SMART patients and SI individuals revealed more pronounced alterations. The longitudinal NK cell analysis of SMART patients further indicates that while overall NK cell frequencies remained stable, surgery was associated with distinct changes in NK cell subset distribution and marker expression during postoperative follow-up, underscoring the impact of surgical intervention on NK cell phenotypes. In more detail, we observed an increased proportion of CD56^bright^ NK cells suggesting a shift towards a more immunoregulatory NK cell phenotype following surgery [66]. The concurrent downregulation of NKp30, NKG2D, DNAM-1, PD-1, TIGIT, and TIM-3 indicates postoperative modulation of NK cell phenotypes. However, the functional implications of these changes remain to be determined. Previous studies have linked these alterations with longer-lasting NK cell activation states [67–69]. Changes in chemokine receptor expression further suggest that surgery might be associated with modulation of NK cell chemokine receptor profiles [13, 49–52].

Some limitations should be acknowledged when interpreting these findings. Acute fracture and subsequent surgical intervention represent possible confounders that may influence NK cell phenotypes independently of frailty. As it was previously shown that CMV serostatus has an impact on NK cell phenotype [70], we need to acknowledge that no CMV serostatus data were available in our cohort. Moreover, subgroup analyses, functional NK cell assays and SIM analyses were performed only in a small cohort, limiting statistical power and generalizability and potentially introducing residual selection bias. Therefore, the observed correlations should be considered exploratory and interpreted as associative rather than mechanistic.

## Conclusion

In conclusion, our findings demonstrate that older fracture patients exhibit reduced circulating NK cell frequencies, altered NK cell phenotypes, and a distinct proinflammatory cytokine profile compared with healthy older adults. These alterations appear to be more closely associated with the acute fracture and hospitalization setting than with frailty status alone. The observed changes in inflammatory mediators and chemokine receptor expression raise the possibility that inflammatory processes influence NK cell biology in this context. However, the underlying mechanisms and their clinical implications remain to be determined.

## Supplementary Information

Supplementary Material 1.

## Data Availability

The datasets used and/or analysed during the current study are available from the corresponding author on reasonable request.

## References

[1] Guo J, Huang X, Dou L, Yan M, et al. Aging and aging-related diseases: from molecular mechanisms to interventions and treatments. Sig Transduct Target Ther. 2022;1:391. 10.1038/s41392-022-01251-0 PMC9755275 36522308

[2] David E, Bloom DLL. The global demography of aging: Facts, explanations, future. Handbook Econ Popul Aging. 2016;1:3–56.

[3] Nikolich-Žugich J. The twilight of immunity: emerging concepts in aging of the immune system. Nat Immunol. 2017;1:10. 10.1038/s41590-017-0006-x 29242543

[4] Deng Y, Zheng Z, Cheng S, Lin Y, et al. The factors associated with nosocomial infection in elderly hip fracture patients: gender, age, and comorbidity. Int Orthop. 2021;12:3201–9. 10.1007/s00264-021-05104-3 34350473

[5] Kim DH, Rockwood K. Frailty in older adults. N Engl J Med. 2024;6:538–48. 10.1056/NEJMra2301292 PMC11634188 39115063

[6] Fried LP, Tangen CM, Walston J, Newman AB, et al. Frailty in older adults: evidence for a phenotype. J Gerontol A Biol Sci Med Sci. 2001;3:146–56. 10.1093/gerona/56.3.m146 11253156

[7] Lin H, McBride RL, Hubbard RE. Frailty and anesthesia - risks during and post-surgery. Local Reg Anesth. 2018;11:61–73. 30323657 10.2147/LRA.S142996 PMC6178933

[8] Robinson TN, Walston JD, Brummel NE, Deiner S, et al. Frailty for surgeons: review of a national institute on aging conference on frailty for specialists. J Am Coll Surg. 2015;6:1083–92. 10.1016/j.jamcollsurg.2015.08.428 PMC4673051 26422746

[9] McIsaac DI, Bryson GL, van Walraven C. Association of frailty and 1-year postoperative mortality following major elective noncardiac surgery: A population-based cohort study. JAMA Surg. 2016;6:538–45. 10.1001/jamasurg.2015.5085 26791334

[10] Riemann L, Gutierrez R, Odak I, Barros-Martins J, et al. Integrative deep immune profiling of the elderly reveals systems-level signatures of aging, sex, smoking, and clinical traits. EBioMedicine. 2025;112:105558. 39862806 10.1016/j.ebiom.2025.105558 PMC11873576

[11] Howlett SE, Rutenberg AD, Rockwood K. The degree of frailty as a translational measure of health in aging. Nat Aging. 2021;8:651–65. 10.1038/s43587-021-00099-3 37117769

[12] Brauning A, Rae M, Zhu G, Fulton E, et al. Aging of the immune system: focus on natural killer cells phenotype and functions. Cells. 2022;6:1017. 10.3390/cells11061017 PMC8947539 35326467

[13] Ran G, Yq L, Tian L, Zhang T, et al. Natural killer cell homing and trafficking in tissues and tumors: From biology to application. Sig Transduct Target Ther. 2022;1:205. 10.1038/s41392-022-01058-z PMC9243142 35768424

[14] Chen S, Zhu H, Jounaidi Y. Comprehensive snapshots of natural killer cells functions, signaling, molecular mechanisms and clinical utilization. Sig Transduct Target Ther. 2024;1:302. 10.1038/s41392-024-02005-w PMC11544004 39511139

[15] Abel AM, Yang C, Thakar MS, Malarkannan S. Natural killer cells: development, maturation, and clinical utilization. Front Immunol. 2018;1869:11–21. 10.3389/fimmu.2018.01869 PMC6099181 30150991

[16] Smyth MJ, Cretney E, Kelly JM, Westwood JA, et al. Activation of NK cell cytotoxicity. Mol Immunol. 2005;4:501–10. 10.1016/j.molimm.2004.07.034 15607806

[17] Gounder SS, Abdullah BJJ, Radzuanb NEIBM, Zain FDBM, et al. Effect of aging on NK cell population and their proliferation at ex vivo culture condition. Anal Cell Pathol (Amst). 2018;7871814:1–6. 10.1155/2018/7871814 PMC6098903 30175033

[18] Guo Z, Wu F, Chen Y, Xu J, et al. Phenotypes, mechanisms, and therapeutic strategies of natural killer cell immunosenescence. Immunity Ageing. 2025;1:38. 10.1186/s12979-025-00534-8 PMC12538774 41121385

[19] Roesner LM, Gupta MK, Kopfnagel V, van Unen N, et al. The RESIST senior individuals cohort: Design, participant characteristics and aims. GeroScience. 2024;4:6101–2. 10.1007/s11357-024-01380-0 PMC12397005 39436551

[20] Ho D, Imai K, King G, Stuart EA. MatchIt: Nonparametric preprocessing for parametric causal inference. J Stat Softw. 2011;42:1–28.

[21] Mahoney FI, Barthel DW. Functional evaluation: the barthel index. Md State Med J. 1965;14:61–5. 14258950

[22] Hamilton GF, McDonald C, Chenier TC. Measurement of grip strength: validity and reliability of the sphygmomanometer and jamar grip dynamometer. J Orthop Sports Phys Ther. 1992;5:215–9. 10.2519/jospt.1992.16.5.215 18796752

[23] Morley JE, Vellas B, van Kan GA, Anker SD, Bauer JM, Bernabei R, et al. Frailty consensus: a call to action. JAMDA. 2013;6:392–7. 10.1016/j.jamda.2013.03.022 PMC4084863 23764209

[24] Parker MJ, Palmer CR. A new mobility score for predicting mortality after hip fracture. J Bone Joint Surg Br. 1993;5:797–8. 10.1302/0301-620X.75B5.8376443 8376443

[25] Lawton MP, Brody EM. Assessment of older people: self-maintaining and instrumental activities of daily living. Gerontologist. 1969;3:179–86. 5349366

[26] Folstein MF, Folstein SE, McHugh PR. “Mini-mental state”. A practical method for grading the cognitive state of patients for the clinician. J Psychiatr Res. 1975;3:189–98. 10.1016/0022-3956(75)90026-6 1202204

[27] Brink TL, Yesavage JA, Lum O, Heersema PH, et al. Screening tests for geriatric depression. Clin Gerontol. 1982;1:37–43.

[28] Nasreddine ZS, Phillips NA, Bédirian V, Charbonneau S, et al. The montreal cognitive assessment, MoCA: a brief screening tool for mild cognitive impairment. J Am Geriatr Soc. 2005;4:695–9. 10.1111/j.1532-5415.2005.53221.x 15817019

[29] Beck AT, Ward CH, Mendelson M, Mock J, et al. An inventory for measuring depression. Arch Gen Psychiatry. 1961;6:561–71. 10.1001/archpsyc.1961.01710120031004 13688369

[30] Niehaus CE, Strunz B, Cornillet M, Falk CS, et al. MAIT cells are enriched and highly functional in ascites of patients with decompensated liver cirrhosis. Hepatology. 2020;4:1378–93. 10.1002/hep.31153 32012321

[31] Becht E, McInnes L, Healy J, Dutertre C, et al. Dimensionality reduction for visualizing single-cell data using UMAP. Nat Biotechnol. 2018;37:38–44. 10.1038/nbt.4314 30531897

[32] Levine JH, Simonds EF, Bendall SC, Davis KL, et al. Data-driven phenotypic dissection of AML reveals progenitor-like cells that correlate with prognosis. Cell. 2015;1:184–97. 10.1016/j.cell.2015.05.047 PMC4508757 26095251

[33] Tang F, Tie Y, Tu C, Wei X. Surgical trauma-induced immunosuppression in cancer: recent advances and the potential therapies. Clin Transl Med. 2020;1:199–223. 10.1002/ctm2.24 PMC7240866 32508035

[34] Mailliez A, Guilbaud A, Puisieux F, Dauchet L, et al. Circulating biomarkers characterizing physical frailty: CRP, hemoglobin, albumin, 25OHD and free testosterone as best biomarkers. Results of a meta-analysis. Exp Gerontol. 2020;139:111014. 32599147 10.1016/j.exger.2020.111014

[35] Picca A, Coelho-Junior HJ, Calvani R, Marzetti E, et al. Biomarkers shared by frailty and sarcopenia in older adults: A systematic review and meta-analysis. Ageing Res Rev. 2022;73:101530. 34839041 10.1016/j.arr.2021.101530

[36] Steinmeyer Z, Delpierre C, Soriano G, Steinmeyer A, et al. Hemoglobin concentration; a pathway to frailty. BMC Geriatr. 2020;1:202. 10.1186/s12877-020-01597-6 PMC7291509 32527230

[37] Ranieri P, Rozzini R, Franzoni S, Barbisoni P, et al. Serum cholesterol levels as a measure of frailty in elderly patients. Exp Aging Res. 1998;2:169–79. 10.1080/036107398244300 9555569

[38] Zhang L, Yang P, Yin F, Zhang J, et al. Association between frailty and hypoproteinaemia in older patients: Meta-analysis and systematic review. BMC Geriatr. 2024;1:689. 10.1186/s12877-024-05275-9 PMC11329991 39154175

[39] Lv Y, Mao C, Gao X, Yin Z, et al. Triglycerides paradox among the oldest old: “the lower the better?” J Am Geriatr Soc. 2019;4:741–8. 10.1111/jgs.15733 PMC6458070 30628728

[40] Duggal NA, Upton J, Phillips AC, Hampson P, et al. NK cell immunesenescence is increased by psychological but not physical stress in older adults associated with raised cortisol and reduced perforin expression. Age (Dordr). 2015;1:9748. 10.1007/s11357-015-9748-2 PMC4320126 25663421

[41] Qi C, Liu Q. Natural killer cells in aging and age-related diseases. Neurobiol Dis. 2023;183:106156. 37209924 10.1016/j.nbd.2023.106156

[42] Yao Q, Su Z, Luo L, Luo H. Frailty assessment and NK cell function in multiple myeloma: a comprehensive analysis. Clin Lab. 2025;71:1. 10.7754/Clin.Lab.2024.241121 40663087

[43] Kaur K, Jewett A. Decreased surface receptors, function, and suboptimal osteoclasts-induced cell expansion in natural killer (NK) cells of elderly subjects. Aging (Albany NY). 2025;3:798–821. 10.18632/aging.206226 PMC11984426 40146570

[44] Tran Van Hoi E, Santegoets SJ, Mooijaart SP, Van Heemst D, et al. Blood based immune biomarkers associated with clinical frailty scale in older patients with melanoma receiving checkpoint inhibitor immunotherapy. Immun Ageing. 2024;1:83. 10.1186/s12979-024-00463-y PMC11600645 39593063

[45] Yu L, Liu X, Wang X, Yan F, et al. TIGIT + TIM-3 + NK cells are correlated with NK cell exhaustion and disease progression in patients with hepatitis B virus-related hepatocellular carcinoma. Oncoimmunology. 2021;1:1942673. 10.1080/2162402X.2021.1942673 PMC8244763 34249476

[46] Bi J, Tian Z. NK cell exhaustion. Front Immunol. 2017;8:760. 28702032 10.3389/fimmu.2017.00760 PMC5487399

[47] Gallois A, Silva I, Osman I, Bhardwaj N. Reversal of natural killer cell exhaustion by TIM-3 blockade. Oncoimmunology. 2014;12:e946365. 10.4161/21624011.2014.946365 PMC4353130 25964857

[48] Duan S, Guo W, Xu Z, He Y, et al. Natural killer group 2D receptor and its ligands in cancer immune escape. Mol Cancer. 2019;1:29. 10.1186/s12943-019-0956-8 PMC6391774 30813924

[49] Qin S, Rottman JB, Myers P, Kassam N, et al. The chemokine receptors CXCR3 and CCR5 mark subsets of T cells associated with certain inflammatory reactions. J Clin Invest. 1998;4:746–54. 10.1172/JCI1422 PMC508621 9466968

[50] Liu Y, Zhu L, Zhang Z, Liu T, et al. C-C chemokine receptor 5 is essential for conventional NK cell trafficking and liver injury in a murine hepatitis virus-induced fulminant hepatic failure model. J Transl Med. 2023;1:865. 10.1186/s12967-023-04665-8 PMC10685630 38017505

[51] Kim J, Kim JS, Lee HK, Kim HS, et al. CXCR3-deficient natural killer cells fail to migrate to B16F10 melanoma cells. Int Immunopharmacol. 2018;63:66–73. 30075430 10.1016/j.intimp.2018.07.026

[52] Lachota M, Zielniok K, Palacios D, Kanaya M, et al. Mapping the chemotactic landscape in NK cells reveals subset-specific synergistic migratory responses to dual chemokine receptor ligation. EBioMedicine. 2023;96:104811. 37741009 10.1016/j.ebiom.2023.104811 PMC10520535

[53] Ware CF, Croft M, Neil GA. Realigning the LIGHT signaling network to control dysregulated inflammation. J Exp Med. 2022;7:e20220236. 10.1084/jem.20220236 PMC9130030 35604387

[54] Kim JH, Kim K, Kim I, Seong S, et al. The MCP-3/Ccr3 axis contributes to increased bone mass by affecting osteoblast and osteoclast differentiation. Exp Mol Med. 2024;11:2465–74. 10.1038/s12276-024-01344-6 PMC11612511 39482538

[55] Guo X, Zhou R, Tian G, Shi W, Lu J, Li R. Genetic insights into the causal linkage between systemic inflammatory regulators and frailty. Cytokine. 2024;184:156791. 39447338 10.1016/j.cyto.2024.156791

[56] Kamat K, Krishnan V, Dorigo O. Macrophage-derived CCL23 upregulates expression of T-cell exhaustion markers in ovarian cancer. Br J Cancer. 2022;6:1026–33. 10.1038/s41416-022-01887-3 PMC9470573 35750747

[57] Beamer B, Hettrich C, Lane J. Vascular endothelial growth factor: an essential component of angiogenesis and fracture healing. HSS J. 2010;1:85–94. 10.1007/s11420-009-9129-4 PMC2821499 19763695

[58] Zhen R, Yang J, Wang Y, Li Y, et al. Hepatocyte growth factor improves bone regeneration via the bone morphogenetic protein-2-mediated NF-κB signaling pathway. Mol Med Rep. 2018;4:6045–53. 10.3892/mmr.2018.8559 29436622

[59] Nicola NA, Babon JJ. Leukemia inhibitory factor (LIF). Cytokine Growth Factor Rev. 2015;5:533–44. 10.1016/j.cytogfr.2015.07.001 PMC4581962 26187859

[60] Vadaq N, van de Wijer L, van Eekeren LE, Koenen H, et al. Targeted plasma proteomics reveals upregulation of distinct inflammatory pathways in people living with HIV. iScience. 2022;10:105089. 10.1016/j.isci.2022.105089 PMC9494231 36157576

[61] Köttstorfer J, Thomas A, Gregori M, Kecht M, et al. Are OPG and RANKL involved in human fracture healing? J Orthop Res. 2014;12:1557–61. 10.1002/jor.22723 25212894

[62] Schett G, Kiechl S, Redlich K, Oberhollenzer F, et al. Soluble RANKL and risk of nontraumatic fracture. JAMA. 2004;9:1108–13. 10.1001/jama.291.9.1108 14996780

[63] Pansarasa O, Mimmi MC, Davin A, Giannini M, et al. Inflammation and cell-to-cell communication, two related aspects in frailty. Immun Ageing. 2022;1:49. 10.1186/s12979-022-00306-8 PMC9598012 36289502

[64] Molofsky AB, Savage AK, Locksley RM. Interleukin-33 in tissue homeostasis, injury, and inflammation. Immunity. 2015;6:1005–19. 10.1016/j.immuni.2015.06.006 PMC4471869 26084021

[65] Su C, Zhao K, Xia H, Xu Y. Peripheral inflammatory biomarkers in Alzheimer’s disease and mild cognitive impairment: a systematic review and meta-analysis. Psychogeriatr. 2019;4:300–9. 10.1111/psyg.12403 30790387

[66] Cooper MA, Fehniger TA, Turner SC, Chen KS, et al. Human natural killer cells: a unique innate immunoregulatory role for the CD56(bright) subset. Blood. 2001;10:3146–51. 10.1182/blood.v97.10.3146 11342442

[67] Market M, Tennakoon G, Scaffidi M, Cook DP, et al. Preventing surgery-induced NK cell dysfunction using anti-TGF-β immunotherapeutics. Int J Mol Sci. 2022;23:14608. 36498937 10.3390/ijms232314608 PMC9737532

[68] Cao Y, Wang X, Jin T, Tian Y, et al. Immune checkpoint molecules in natural killer cells as potential targets for cancer immunotherapy. Sig Transduct Target Ther. 2020;1:250. 10.1038/s41392-020-00348-8 PMC7596531 33122640

[69] Farhat M, Croft W, Parry HM, Verma K, et al. PD-1 expression contributes to functional impairment of NK cells in patients with B-CLL. Leukemia. 2024;8:1813–7. 10.1038/s41375-024-02271-1 PMC11286510 38724674

[70] Leng SX, Margolick JB. Aging, sex, inflammation, frailty, and CMV and HIV infections. Cell Immunol. 2020;348:104024. 10.1016/j.cellimm.2019.104024 PMC7002257 31843200

