# Supplementary material for: Reduced NK cell frequency in older patients with evolving fractures is linked to a distinct inflammatory cytokine profile
Source: Immun Ageing. 2026 Jul 24;23:24. doi: 10.1186/s12979-026-00585-5 (PMC13397730; doi:10.1186/s12979-026-00585-5)
Supplement: Supplementary file 1 — Supplementary Material 1. [file 12979_2026_585_MOESM1_ESM.docx]

**Supplemental material**

**Reduced NK cell frequency in older patients with evolving fractures is linked to a distinct inflammatory cytokine profile**

Julia P. Moch, Jolane Kappes, Rodrigo Gutierrez Jauregui, Hagen Schmaus, Malou-Sophie Dietrich, Daniel A. Thies, Lennart M. Roesner, Thomas Werfel, Reinhold Förster, Dorothee von Witzendorff, Jennifer Debarry, Marcel Winkelmann, Swantje Oberthür, Jan-Dierk Clausen, Manfred Gogol, Markus Cornberg, Anke R.M Kraft*, Christian Niehaus*

**Table of contents**

Supplementary Table 1.1

Supplementary Table 1.2

Supplementary Table 1.3

Supplementary Table 2.1

Supplementary Table 2.2

Supplementary Table 2.3

Supplementary Table 2.4

Supplementary Table 2.5

Supplementary Figure legends

Supplementary Figure 1

Supplementary Figure 2

Supplementary Figure 3

Supplementary Figure 4

Supplementary Figure 5

Supplementary Figure 6

Supplementary Figure 7

**Supplementary Table 1.1.** Baseline characteristics and geriatric assessment of matched SMART patients and SI individuals.

|  | **SMART COHORT (n= 24)** | **SI COHORT (n= 24)** |  | **p value**  **Reference ranges** |
| --- | --- | --- | --- | --- |
| Age (years), median (IQR) | 84.5 (77.0-87.8) | 84.0 (77.0-87.0) |  | 0.8832 |
| Male/female, n (%) | 9/15 (37.5/62.5) | 9/15 (37.5/62.5) |  | >0.9 |
| BMI (kg/m^2^) median (IQR) | 22.8 (20.3-27.3) | 25.5 (23.9-28.1) | 18.5-24.9 | 0.109 |
| **Geriatric assessments** |  |  |  |  |
| Grip strength (kg), median (IQR) | 18.7 (10.1-23.3) | 30.4 (26.4-37.5) | * | **<0.0001** |
| Barthel scale, median (IQR) | 82.5 (66.3-95.0) | 100.0 (100.0-100.0) | 0-100 | **<0.0001** |
| **Hematological parameters** |  |  |  |  |
| Erythrocytes (Mio/µL), median (IQR) | 4.1 (3.7-4.4) | 4.4 (3.9-4.6) | * | **0.0392** |
| Hemoglobin (g/dL), median (IQR) | 12.5 (11.3-14.1) | 13.4 (12.3-14.1) | * | 0.1012 |
| Hematocrit (%), median (IQR) | 36.2 (33.7-39.8) | 38.7 (35.5-42.1) | * | **0.0345** |
| RDW (%), median (IQR) | 13.5 (12.8-14.4) | 13.9 (12.8-14.6) | 11.5-14.5 | 0.7943 |
| MCV (fL), median (IQR) | 88.3 (86.7-91.6) | 89.7 (86.1-92.9) | 80-100 | 0.7160 |
| MCH (pg), median (IQR) | 30.5 (29.8-32.0) | 30.4 (29.6-31.6) | 27-34 | 0.5715 |
| MCHC (g/dL), median (IQR) | 34.6 (33.6-35.3) | 34.4 (33.8-35.3) | 32-36 | 0.9641 |
| Leukocytes (K/µL), median (IQR) | 8.9 (7.3-10.2) | 5.9 (4.6-7.8) | 4.0-10.0 | **0.0003** |
| Platelets (K/µL), median (IQR) | 221.0 (191.8-267.5) | 224.0 (199.5-244.0) | 150-400 | 0.6657 |
| MPV (fL), median (IQR) | 10.0 (9.4-10.2) | 10.7 (9.8-11.2) | 7.5-12.0 | **0.0138** |
| Platelets >12fL (%), median (IQR) | 19.7 (17.1-22.3) | 30.6 (22.3-34.5) | 15.0-35.0 | **<0.0001** |
| PDW (fL), median (IQR) | 9.8 (9.3-11.1) | 12.2 (10.5-13.5) | 9.0-17.0 | **0.0008** |
| **Inflammation** |  |  |  |  |
| CRP (mg/L), median (IQR) | 5.85 (2.0-12.5) | 1.6 (0.7-2.7) | <5 | **0.0003** |
| **Kidney function** |  |  |  |  |
| Creatinine (µmol/L), median (IQR) | 80.5 (67.8-101.0) | 85.0 (70.8-104.0) | * | 0.5634 |
| eGFR (Creatinine) (mL/min/1), median (IQR) | 65.5 (47.3-77.8) | 59.0 (49.0-77.8) | * | 0.9288 |
| **Enzymes** |  |  |  |  |
| ALT (U/L), median (IQR) | 17.0 (13.0-23.0) | 18.0 (14.0-21.0) | * | 0.6534 |
| AST (U/L), median (IQR) | 27.0 (21.0-36.0) | 24.0 (22.0-26.0) | * | 0.1722 |
| **Clinical chemistry** |  |  |  |  |
| HDL-C (mg/dL), median (IQR) | 68.0 (44.0-75.0) | 54.5 (54.5-72.3) | * | 0.8247 |
| LDL-C (mg/dL), median (IQR) | 86.0 (31.0-129.5) | 116.0 (91.3-141.3) | * | 0.0891 |
| Cholesterol total (mg/dL), median (IQR) | 174.0 (143.0-226.0) | 201.0 (164.0-222.3) | <200 | 0.2085 |
| Uric acid (µmol/L), median (IQR) | 317.5 (232.5-393.0) | 337.0 (265.5-414.0) | * | 0.4363 |
| Total protein (g/L), median (IQR) | 64.0 (63.0-69.5) | 68.0 (66.0-71.0) | 64-83 | **0.0333** |
|  |  |  |  |  |

**Supplementary Table 1.1.** Baseline characteristics and geriatric assessment of matched SMART patients and SI individuals.

SMART patients (n= 24) were compared with matched SI individuals (n= 24). Matching by age and gender was performed using R Studio 4.3.2 (MatchIt package). Continuous variables are presented as median with IQR (Q1-Q3). Categorical values are presented as frequencies and percentages. Level of significance: p<0.05. Mann-Whitney U test was performed for comparison of non-normally distributed values, and an unpaired t-test was used for normally distributed values. ALT, alanine aminotransferase; AST, aspartate aminotransferase; BMI, Body mass index; CRP, C-reactive protein; eGFR, estimated Glomerular Filtration Rate; HDL-C, high-density lipoprotein cholesterol; LDL-C, low-density lipoprotein cholesterol; MCV, mean corpuscular volume; MCH, mean corpuscular hemoglobin; MCHC, mean corpuscular hemoglobin concentration; MPV, mean platelet volume; N.d. not determined; PDW, platelet distribution width; RDW, red cell distribution width. Reference ranges are based on standard adult clinical laboratory reference intervals but may vary between laboratories and analytical methods; * Reference ranges are sex dependent.

**Supplementary Table 1.2.** Geriatric assessment of SMART patients.

| **Geriatric assessment** | **Result** | **Range** |
| --- | --- | --- |
| Clinical frailty scale, median (IQR) | 5.0 (2.3-6.0) | 1-9 |
| Parker Mobility Score, median (IQR) | 6.0 (4.3-9.0) | 0-9 |
| Mini Mental Status Examination, median (IQR) | 26.0 (22.5-30.0) | 0-30 |
| IADL (Lawton scale), median (IQR) | 6.0 (4.3-8.0) | 0-8 |
| Geriatric depression scale, median (IQR) | 3.0 (2.0-4.0) | 0-15 |

**Supplementary Table 1.3.** Geriatric assessment of SI individuals.

| **Geriatric assessment** | **Result** | **Range** |
| --- | --- | --- |
| Beck’s Depression Inventory, median (IQR) | 1.0 (0.0-2.0) | 0-63 |
| Montreal Cognitive Assessment, median (IQR) | 24.0 (23.0-28.0) | 0-30 |

**Supplementary Table 2.1.** Antibodies used for flow cytometry; Adaptive immune cell antibody panel.

| **Specificity** | **Fluorochrome** | **Clone** | **Company** |
| --- | --- | --- | --- |
| CD16 | BUV496 | 3G8 | BD |
| CD4 | BUV563 | RPA-T4 | BD |
| CD56 | BUV737 | NCAM16.2 | BD |
| CD20 | BV570 | L243 | BioLegend |
| CXCR3 | BV650 | G025H7 | BioLegend |
| CCR6 | BV711 | G034E3 | BioLegend |
| CXCR5 | BV750 | RF8B2 | BD |
| PD-1 | FITC | NAT105 | BioLegend |
| CD3 | AF532 | UCHT1 | Invitrogen |
| CD8 | SparkBlue550 | SK1 | BioLegend |
| CD19 | PerCP | HIB19 | BioLegend |
| CD14 | BB700 | MoP9 | BD |
| CD38 | PerCP-eF710 | HB7 | Invitrogen |
| HLA-DR | PE-Fire810 | L243 | BioLegend |
| γδ-TCR | APC | 11F2 | Miltenyi |
| Viability | Zombie NIR |  | BioLegend |
| Tim3 | APC Fire750 | F38-2E2 | BioLegend |

**Supplementary Table 2.2.** Antibodies used for flow cytometry; Innate immune cell antibody panel.

| **Specificity** | **Fluorochrome** | **Clone** | **Company** |
| --- | --- | --- | --- |
| CD45 | BUV395 | HI30 | BD |
| CD16 | BUV496 | 3G8 | BD |
| CD14 | BUV563 | MoP9 | BD |
| CD11c | BUV661 | B-ly6 | BD |
| CD56 | BUV737 | NCAM16.2 | BD |
| CCR5 | BV510 | J418F1 | BioLegend |
| HLA-DR | BV570 | L243 | BioLegend |
| CD3 | AF532 | UCHT1 | Invitrogen |
| NKG2D | PE | 1D11 | BioLegend |
| CD19 | PE-Cy7 | SJ25C1 | BD |
| Viability | Zombie NIR |  | BioLegend |

**Supplementary Table 2.3.** Antibodies used for flow cytometry; Cytotoxic and activation marker antibody panel.

| **Specificity** | **Fluorochrome** | **Clone** | **Company** |
| --- | --- | --- | --- |
| Granzyme K | PerCP-eFluor 710 | G3H69 | Thermofisher |
| Granzyme B | PE-Cy7 | QA16A02 | BioLegend |
| CCR7 | PE/Fire 810 | G043H7 | BioLegend |
| HLA-DR | FITC | G46-6 | BD |
| γδ-TCR | PE | 11F2 | Miltenyi |
| NKG2D | PE/Dazzle 594 | 1D11 | BioLegend |
| CD25 | PE-Cy5 | M-A251 | BD |
| CD14 | APC | REA599 | Miltenyi |
| Granulysin | AF647 | DH2 | BioLegend |
| CXCR3 | AF700 | G025H7 | BioLegend |
| CD161 | APC-Vio770 | HP-3G10 | Miltenyi |
| CD19 | APC/Fire 810 | HIB19 | BioLegend |
| TCR Vα7.2 | BUV615 | OF-5A12 | BD OptiBuild |
| CD3 | BUV661 | UCHT1 | BD Horizon |
| CD69 | BUV737 | FN50 | BD |
| CD8 | BUV805 | SK1 | BD Horizon |
| CD38 | BUV395 | HB7 | BD Horizon |
| LiveDead | LD Blue |  | Thermofisher |
| CD16 | BUV496 | 3G8 | BD Horizon |
| CD56 | BUV563 | NCAM16.2 | BD Horizon |
| CXCR6 | BV421 | K041E5 | BioLegend |
| CD45RA | BV605 | HI100 | BioLegend |
| CD127 | BV650 | A019D5 | BioLegend |
| CD103 | BV711 | Ber-ACT8 | BD |
| PD-1 | BV786 | EH12.2H7 | BioLegend |
| Perforin | VioBlue | delta G9 | Miltenyi |
| CX3CR1 | BV480 | 2A9-1 | BD OptiBuild |
| Vδ1 | BV510 | REA173 | BioLegend |
| CD4 | BV570 | RPA-T4 | BioLegend |

**Supplementary Table 2.4.** Antibodies used for flow cytometry; NK cell focused follow-up analysis.

| **Specificity** | **Fluorochrome** | **Clone** | **Company** |
| --- | --- | --- | --- |
| CXCR3 | BV785 | G025H7 | BioLegend |
| CXCR6 | BV480 | 13B 1E5 | BD |
| CD161 | BUV395 | HP-3G10 | BD |
| CD14 | BUV496 | M5E2 | BD |
| CD16 | BUV563 | 3G8 | BD |
| TIGIT | BUV615 | 741182 | BD |
| TIM-3 | BUV737 | 7D3 | BD |
| NKp30 | BUV805 | p30-15 | BD |
| PD-1 | BV421 | Nat105 | BioLegend |
| CD57 | BV605 | QA17A04 | BioLegend |
| CD3 | BV650 | OKT3 | BioLegend |
| DNAM-1 | BV711 | 11A8 | BioLegend |
| CD69 | BV750 | FN50 | BioLegend |
| CD158 | FITC | HP-MA4 | BioLegend |
| NKG2C | PE | S19005E | BioLegend |
| NKG2D | PE-CF94 | 1D11 | BD |
| CD19 | PE-Fire640 | HIB19 | BioLegend |
| CD8a | PerCP | RPA-T8 | BioLegend |
| CD4 | PE/Dazzle594 | RPA-T4 | BioLegend |
| GPR56 | PE/Cy7 | CG4 | BioLegend |
| CD56 | AF647 | 5.1H11 | BioLegend |
| NKG2A | APC/Fire750 | S19004C | BioLegend |
| CD38 | APC/Fire810 | HIT2 | BioLegend |
| Viability | ZombieAqua |  | BioLegend |

**Supplementary Table 2.5** Antibodies used for flow cytometry; functional NK cell analysis.

| **Specificity** | **Fluorochrome** | **Clone** | **Company** |
| --- | --- | --- | --- |
| IFNy | FITC | B27 | BioLegend |
| NKG2D | PE | 1D11 | BD |
| Granzyme B | PE-Dazzle 594 | QA16A02 | BioLegend |
| Granzyme K | PECy7 | GM26E7 | BioLegend |
| CD56 | PerCP/Cy 5.5 | MEM-188 | BioLegend |
| Granulysin | APC | DH2 | BioLegend |
| CD3 | APC/Fire 750 | UCHT1 | BioLegend |
| Perforin | BV421 | dG9 | BioLegend |
| CD16 | BV510 | 3G8 | BioLegend |
| CD107a | BV605 | H4A3 | BioLegend |
| CD8 | BV650 | SK1 | BioLegend |
| PD-L1 | BV711 | 29E.2A3 | BioLegend |
| CD38 | BV786 | HIT2 | BD |
| CD14 | AlexaFlour 700 | M5E2 | BioLegend |
| CD19 | AlexaFlour 700 | HIB19 | BioLegend |

**Supplementary Figure 1. Gating strategy to identify effector cells and NK cell phenotype.**

Flow cytometric data were manually gated using FlowJo software. Lymphocytes were gated based on FSC/SSC after exclusion of doublets and dead cells. NK cells were gated as live, singlet, CD3^-^CD56⁺ lymphocytes and were further subdivided into CD56^dim^ and CD56^bright^ subsets. NK-T like cells were defined as CD3⁺CD56⁺, while CD4⁺, CD8⁺, and γδ T cells were all gated on CD3⁺ lymphocytes. B cells were identified as CD19⁺CD20⁺ cells.

**Supplementary Figure 2. Sex-specific differences in effector cell frequencies in SMART patients and SI individuals.**

Sex-stratified frequencies of effector cell subsets in SMART patients (A) and SI individuals (B). SMART: 7 males/17 females; SI: 11 males/12 females. Bars represent mean ± SD. Data were tested for normality, and comparisons were performed using either an unpaired t-test or a Mann–Whitney U test, as appropriate.

**Supplementary Figure 3. Correlation matrix of assessment results, laboratory values, and effector cell frequencies in SMART patients.**

Correlation matrix of clinical markers, laboratory values, and effector cell frequencies in SMART patients. Spearman’s rank correlation coefficients (ρ) are shown, with positive (red) and negative (blue) correlations scaled by color intensity. Level of significance was defined as p<0.05 (*). ALT, alanine aminotransferase; AST, aspartate aminotransferase; BMI, Body mass index; CFS, Clinical Frailty Scale; CRP, C-reactive protein; GDS, Geriatric Depression Scale GDS, Geriatric Depression Scale; MMST, Mini Mental Status Test; PMS, Parker Mobility Scale.

**Supplementary Figure 4. Phenotype analysis of CD56^dim^ and CD56^bright^ NK cells in SMART patients compared to SI individuals.**

Expression of activation/exhaustion markers and chemokine receptors on (A) CD56^dim^ NK cells and (B) CD56^bright^ NK cells comparing SMART patients (n= 24) with SI individuals (n= 24). Data represent mean values ± standard deviation (SD) for each marker. Data were assessed for normality and analyzed using unpaired t-tests or Mann-Whitney U tests, as appropriate. p<0.05 (*), p<0.01 (**), p<0.001 (***), p<0.0001 (****). Sex distribution male/female: 9/15 in each cohort.

**Supplementary Figure 5. NK cell phenotype of frail compared with non-frail patients within the SMART cohort.**

Expression of indicated phenotypic markers gated on (A) total NK cells, (B) gated on CD56^dim^ NK cells, and (C) gated on CD56^bright^ NK cells comparing frail (n= 13) and non-frail (n= 11) SMART patients. Data represent mean values ± standard deviation (SD) for each marker. Data were assessed for normality and analyzed using unpaired t-tests or Mann-Whitney U tests, as appropriate. p<0.05 (*), p<0.01 (**), p<0.001 (***), p<0.0001 (****). Sex distribution male/female: 9/15.

**Supplementary Figure 6. Functional NK cell response in frail compared with non-frail patients.**

NK cells from 12 patients within the SMART cohort were stimulated with IL-12, IL-15, and IL-18 to induce cytokine responses. Six patients with a low Clinical Frailty Scale (CFS; 1-2) and six patients with a high CFS (6-9) were selected. Production of pro-inflammatory cytokines and cytotoxic molecules were analyzed and compared with unstimulated controls. Values were tested for normal distribution, and either a paired t-test or a Wilcoxon signed rank test was applied as appropriate. p<0.05 (*), p<0.01 (**), p<0.001 (***), p<0.0001 (****) indicate statistical significance. Sex distribution male/female: 3/9.

**Supplementary Figure 7. Sex-specific differences in cytokine expression in SMART patients and SI individuals.**

Sex-specific differences in cytokine expression in SMART patients and SI individuals. Each cohort included 3 males and 3 females. Bars represent mean ± SD. Data were tested for normality prior to analysis, and comparisons were performed using either an unpaired t-test or a Mann-Whitney U test, as appropriate.
